# A new nairo-like virus associated with human febrile illness in China

**DOI:** 10.1080/22221751.2021.1936197

**Published:** 2021-06-17

**Authors:** Yan-Chun Wang, Zhengkai Wei, Xiaolong Lv, Shuzheng Han, Zedong Wang, Changfa Fan, Xu Zhang, Jianwei Shao, Ying-Hua Zhao, Liyan Sui, Chen Chen, Ming Liao, Bo Wang, Ningyi Jin, Chang Li, Jun Ma, Zhi-Jun Hou, Zhengtao Yang, Zhen Han, Yong Zhang, Junqi Niu, Wei Wang, Youchun Wang, Quan Liu

**Affiliations:** a Department of Emerging Infectious Diseases, The First Hospital of Jilin University, Changchun, People’s Republic of China; b Military Veterinary Institute, Academy of Military Medical Sciences, Changchun, People’s Republic of China; c College of Life Sciences and Engineering, Foshan University, Foshan, People’s Republic of China; d The Second Affiliated Hospital of Inner Mongolia University for the Nationalities, Inner Mongolia General Forestry Hospital, Yakeshi, People’s Republic of China; e National Institutes for Food and Drug Control, Beijing, People’s Republic of China.; f National and Regional Joint Engineering Laboratory for Medicament of Zoonoses Prevention and Control, South China Agricultural University, Guangzhou, People’s Republic of China; g College of Wildlife and Protected Area, Northeast Forestry University, Harbin, People’s Republic of China

**Keywords:** Nairovirus, nairo-like virus, Beiji nairovirus, ticks, patients

## Abstract

Several nairo-like viruses have been discovered in ticks in recent years, but their relevance to public health remains unknown. Here, we found a patient who had a history of tick bite and suffered from a febrile illness was infected with a previously discovered RNA virus, Beiji nairovirus (BJNV), in the nairo-like virus group of the order *Bunyavirales*. We isolated the virus by cell culture assay. BJNV could induce cytopathic effects in the baby hamster kidney and human hepatocellular carcinoma cells. Negative-stain electron microscopy revealed enveloped and spherical viral particles, morphologically similar to those of nairoviruses. We identified 67 patients as BJNV infection in 2017–2018. The median age of patients was 48 years (interquartile range 41–53 years); the median incubation period was 7 days (interquartile range 3–12 days). Most patients were men (70%), and a few (10%) had underlying diseases. Common symptoms of infected patients included fever (100%), headache (99%), depression (63%), coma (63%), and fatigue (54%), myalgia or arthralgia (45%); two (3%) patients became critically ill and one died. BJNV could cause growth retardation, viremia and histopathological changes in infected suckling mice. BJNV was also detected in sheep, cattle, and multiple tick species. These findings demonstrated that the newly discovered nairo-like virus may be associated with a febrile illness, with the potential vectors of ticks and reservoirs of sheep and cattle, highlighting its public health significance and necessity of further investigation in the tick-endemic areas worldwide.

## Introduction

Ticks rank only second to mosquitoes as arthropod vectors of animal and human pathogens, including viruses, bacteria, and parasites [1, 2]. Over the past decade, a large number of new viruses have been discovered in ticks by high-throughput sequencing, including South bay virus, Pustyn virus, Grotenhout virus, Norway nairovirus 1, Gakugsa tick virus, and Beiji nairovirus, which belong to the recently discovered nairo-like virus group in the order *Bunyavirales* [3–8]. However, the relevance of these nairo-like viruses to public health remains to be unveiled.

Monitoring unexplained fever cases in a sentinel hospital is an effective method to identify disease-related pathogens, by which many new tick-borne viruses associated with human diseases, such as severe fever with thrombocytopenia syndrome virus, Alongshan virus (ALSV), and Songling virus (SGLV), have been identified in China [9–11]. Active surveillance for tick-borne diseases led to identification of a patient with an unknown febrile illness in Inner Mongolia Autonomous Region in northeastern China. An investigation was initiated to identify the causative pathogen, revealing a previously discovered nairo-like virus Beiji nairovirus (BJNV) associated with the febrile illness.

## Materials and methods

### Study design and sample collection

The cohort study recruited patients who reported being bitten from ticks in the General Forestry Hospital of Inner Mongolia in 2017–2018, whose blood specimens were collected. The sample size relied on the case number in the hospital during the period. We used a standardized questionnaire to collect data on demography, medical history, and tick exposure of each patient. We obtained the clinical symptoms and laboratory tests from the electronic medical records. We showed these data as they were, without any imputation for the missing data. The patients who were tested positive for viral RNA were included in this study, and the patients who co-infected with other pathogens were excluded. One hundred healthy people who were not bitten from ticks were used as controls; they were matched with the patients in gender and age.

The animal blood samples were collected from sheep and cattle in Hulunbuir, Yakeshi, Inner Mongolia in 2017. Sera were isolated by centrifugation at 1000 *g* for 10 min within 24 h of collection and stored at −80°C until use. Ticks were sampled using flagging vegetation in northeast China in May, 2015, and identified to species by analysis of the morphological traits and 16S ribosomal RNA (rRNA) gene as described previously [12, 13].

### Metagenomic analysis

Viral metagenomics was conducted as described previously [10, 14]. Briefly, blood specimens were centrifuged at 12,000 *g* for 30 min at 4°C, and the supernatants were sequentially filtered through 0.45 and 0.22 µm filters, followed by extraction of viral RNAs. Total RNAs were used for reverse transcription with random primers (5′-GCCGGAGCTCTGCAGATATCNNNNNN-3′), followed by synthesis of double-stranded cDNA (dscDNA) using a Klenow fragment. Sequence-independent single-primer amplification was used for amplification of the dscDNA, and the PCR products were purified and sequenced in the Beijing Genome Institute (BGI, Shenzhen, China).

### Virus isolation

African green monkey kidney (Vero), baby hamster kidney (BHK-21), and human hepatocellular carcinoma (SMMC-7721) cells were used for virus isolation. They were grown in DMEM (Dulbecco’s modified eagle medium) supplemented with 10% fetal bovine serum (FBS), 1 mg/mL streptomycin and 1000 units/ml penicillin and antibiotics.

The blood specimens from patient in the acute stage were centrifuged at 12,000 *g* for 15 min at 4°C. The supernatants were diluted 10 times in DMEM and inoculated onto the confluent monolayer of BHK-21, Vero, and SMMC-7721 cells, respectively. The cells were cultured at 37°C in 5% carbon dioxide, and observed daily for potential viral cytopathic effects (CPE). Three blinded passages of a four-day interval were conducted. The cultural supernatants and pellets were collected to test for the presence of BJNV by a nested RT–PCR. Virus was quantified using fluorescent focus assay, and calculated as fluorescent focus units (FFU) per mL [15]. Electron microscopy of virus was analyzed as described previously [16].

### Molecular detection

Total RNAs were extracted from serum or tissue samples by using QIAamp Viral RNA Mini Kit (Qiagen), followed by synthesis of cDNA using the PrimeScript™ RT reagent Kit with gDNA Eraser (TaKaRa). For the nested PCR, the primers targeted on the N gene of BJNV were designed based on the results of viral metagenomics (Table S1). The 25 μL first-round PCR mixture included 2.5 μL 10× PCR reaction buffer, 50 mM MgCl_2_, 5 pmol of each primer, 1 μL cDNA 0.5 mM dNTP, and 0.1 μL ExTaq Enzyme (Takara). The 25 μL second-round PCR mixture was the same as the first-round PCR mixture with exception of the primers. Amplification was conducted as follows: 94°C for 8 min followed by 30 cycles at 94°C for 40 s, 52–48°C for 40 s, 72°C for 40–60 s, and a final extension at 72°C for 8 min. The PCR products were purified and sequenced.

For real-time PCR analysis, the used primers were based on the L gene of BJNV (Table S1). The 20 μL reaction mixture contained 10 μL 2× SYBR premix Ex TaqII (Takara), 0.4 μM of each primer, 1 μL cDNA. Amplification was performed as follows: 95°C for 40 s followed by 50 cycles at 95°C for 8 s, 60°C for 35 s; and 95°C for 20 s, 60°C for 35 s, 95°C for 20 s for melt curve stage.

### Genetic analysis

The virus genome was obtained through RT-PCR and rapid-amplification of cDNA ends (Table S2). The PCR products were cloned into pMD18-T vector (Takara) and sequenced using the standard sequencing methods. Phylogenetic analyses were conducted using the maximum likelihood method of the Molecular Evolutionary Genetics Analysis (MEGA).

### ELISA analysis

The nucleoprotein gene from BJNV was inserted into pET-30a (Novagen) for prokaryotic expression as described elsewhere [10]. The purified recombinant nucleoprotein was used as a coating antigen (5 ng/µL) in an indirect ELISA for detection BJNV-specific antibodies in patients as described previously [17]. The negative control included 100 serum specimens of healthy individuals. The cutoff value was calculated as the mean optical density (OD) at 450 nm of negative control plus three standard deviations.

### Animal infection

Wild-type C57BL/6 suckling mice were provided by National Institute for Food and Drug Control, Beijing, China, and maintained at a specific pathogen-free facility. Fifty wild-type C57BL/6 suckling mice (50% male and 50% female) were randomly divided into two groups: one group were inoculated by the intracerebral route with 4.2 × 10^2^ FFU of BJNV strain YKS44, and the other group used as control group were administered PBS without virus by the same way. Every five suckling mice were randomly placed in one single cage with their mother, and monitored for 15 days. After administration of BJNV and PBS, the suckling mice that died within 12 h were excluded in statistics. Blood and tissues were collected at indicated time points from euthanized mice for pathological analysis and virus detection. At 15 days post-infection, serum of BJNV-infected mice was collected for virus isolation as described above and quantification of viremia.

The tissue samples, including heart, liver, spleen, lung, kidney, brain, duodenum, and thymus, were collected, fixed in 4% buffered neutral formalin, dehydrated through graded alcohols, embedded in parafﬁn, sliced into pieces (3 μm), and stained with hematoxylin and eosin. Histological changes were observed and captured by a light microscope.

### Data analysis

We presented continuous measurements as mean (standard deviation or standard error) if they are normally distributed, as median (interquartile range, IQR) if they are not, and categorical variables as frequency (percentage). For laboratory results, we also assessed whether the measurements were outside the normal range. We collected the clinical data using Microsoft Excel 2019. All data analyses were conducted with GraphPad Prism software version 5.0 (GraphPad Software Inc., San Diego, USA). The differences between different groups were compared using the Student’s *t* test. The viral infection rates in pooled ticks were determined by maximum likelihood estimation (MLE) in the program PooledInfRate [14]. Two-way analysis of variance was used to analyze the body weight and body temperature changes of infected mice. *p*-values less than 0.05 were considered significant.

## Results

### Virus isolation

In May 2017, a 52-year-old male farmer from Alihe Town of in Inner Mongolia Autonomous Region, China was admitted to the General Forestry Hospital. The patient had a history of tick bite and suffered from a febrile illness with headache seven days after the bite, together with swollen, red, and itchy in bite area. Laboratory tests showed thrombocytosis, leukocytosis and liver function injury in the patient. However, the tick-borne pathogens, such as severe fever with thrombocytopenia syndrome (SFTSV), tick-borne encephalitis virus (TBEV), Alongshan virus (ALSV), *Babesia*, *Anaplasma*, or Lyme disease spirochetes, were tested negative [10, 18–20]. The patient’s blood specimens were used for metagenomics analysis and generated 19 contigs annotated to Beiji nairovirus (BJNV), with approximately 97–99% sequence identity, which has been identified in ticks from Heilongjiang Province, northeastern China (Table S3) [7].

**Figure 1.**
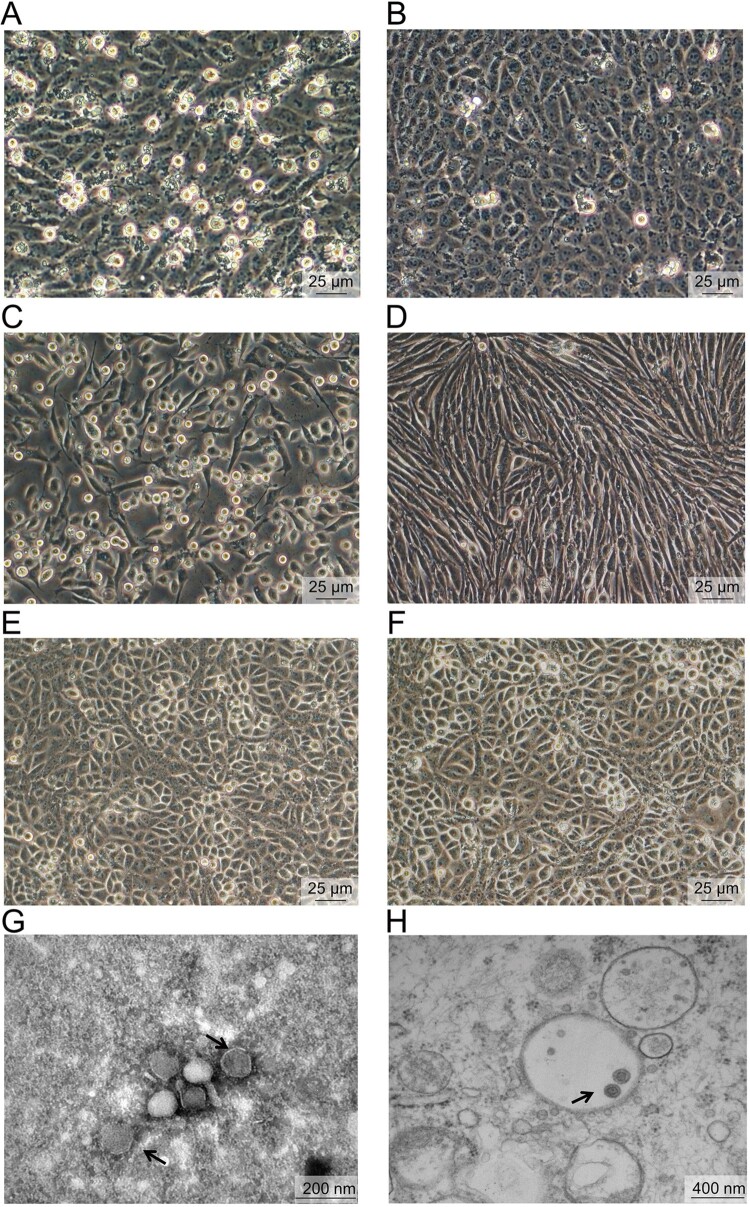
Isolation and electron microscopic examination of BJNV. A, B, SMMC-7721 cells are shown 4 days after infection with BJNV (A) or mock (B); C, D, BHK-21 cells are shown 4 days after infection with BJNV (C) or mock (D); E, F, BHK-21 cells are shown 4 days after infection with BJNV (E) or mock (F); G, H, negatively stained virions purified from infected SMMC-7721 cells (G) and viral particles in the cytoplasmic vacuoles of infected cells (H) are shown (arrows).

We isolated the virus from index patient by cell culture assay. Cytopathic effects (CPE) could be observed by 4–5 days after three passages as evidenced by rounded and detached cells in the infected baby hamster kidney (BHK-21) and human hepatocellular carcinoma (SMMC-7721) cells, but not in Vero cells (Figure 1A–1F). However, the virus, designated YKS44 isolate, could be detected in all three cells with RT-PCR and immunofluorescence assays (Figure S1). We also isolated six additional virus strains from blood samples of other patients during the acute feverish phase of their illness. These viruses could also infect human amniotic (WISH) cells, but could not infect human glioblastoma (U-87MG) and colorectal adenocarcinoma (Caco-2) cells (Figure S1).

Negative-stain electron microscopy revealed enveloped and spherical viral particles, which were similar to the morphology of nairoviruses, with a diameter of approximately 90–100 nm (Figure 1G). Viral particles could be seen in the cytoplasmic vacuoles of infected cells upon transmission electron microscopic analysis (Figure 1H).

### Molecular characterization

**Figure 2.**
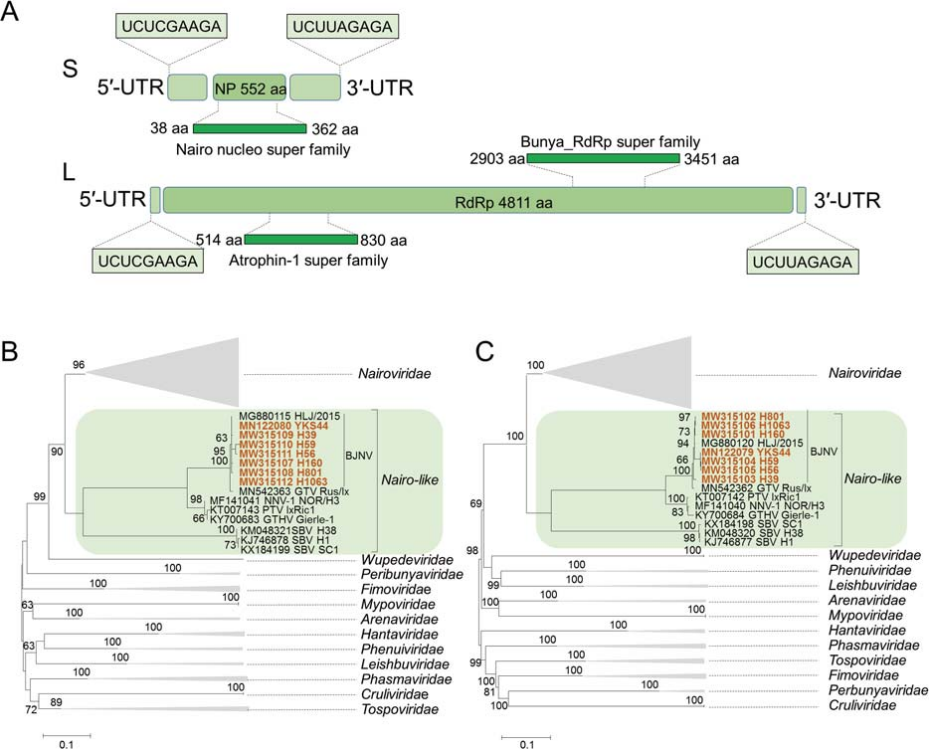
Genome characterization and phylogenetic analysis of BJNV. (A) Schematic genome organization of BJNV. The predicted open reading frames (ORFs) and their super families are shown in boxes; the terminal reverse complementary sequences are indicated (top panel). B, Phylogenetic analysis of BJNV based on the amino acid sequences of the S segment (B), and the L segment (C). The viruses in the order *Bunyavirale* used for phylogenetic analysis are shown in Table S4. The phylogenetic trees were constructed with the Molecular Evolutionary Genetics Analysis software version 5.0 using the Maximum likelihood method with the Jones-Taylor-Thornton model and complete deletion of gaps. Bootstrap testing of 1000 replicates was performed, and the bootstrap values are indicated. Sequences are identified by their GenBank accession numbers, followed by the virus name and strain. The viruses identified in this study are shown in red. The scale bars in each panel indicate 0.1 substitutions per site. BJNV, Beiji nairovirus; GTV, Gakugsa tick virus; NNV-1, Norway nairovirus 1; PTV, Pustyn virus; GTHV, Grotenhout virus; SBV, South Bay virus.

We obtained the complete genome of BJNV using targeted PCR and rapid amplification of complementary DNA ends (RACE), which included the small (S) and large (L) segments of 3719 and 14,851 nucleotides, respectively (Figure 2A). BJNV shared similar genome structure to those of Gakugsa tick virus (GTV), South Bay virus (SBV), Norway nairovirus 1 (NWNV-1), Pustyn virus (PTV), and Grotenhout virus (GTHV) that are grouped into the nairo-like viruses (Figure S2) [4, 6], and had high sequence identity to these newly discovered viruses, especially Gakugsa tick virus (96.6–99.5%) (Tables S5 and S6). Phylogenetic analysis showed that BJNV, together with Gakugsa tick virus (MN542362 and MN542363) identified in Russia, formed a separate clade from other nairo-like viruses, which is genetically related to the members of the family *Nairoviridae* (Figure 2B and 2C).

The typical genome of nairovirus is characterized by the terminal reverse complementary sequences (5′-terminus of UCUCAAAGA and 3′-terminus of AGAGUUUCU) [5]. The sequences of BJNV L and S termini are the same as those of nairoviruses, with an exception of single nucleotide difference at the fifth position (5′-terminus of UCUCGAAGA and 3′-terminus of AGAGAUUCU) (Figure 2A and Figure S3).

The BJNV L segment contained a 14,436-nt ORF encoding a putative 4811-aa RNA-dependent RNA polymerase (RdRp), which is similar to those of NNV-1 and PTV (Figure S2). In BJNV, the catalytic domain was located between the amino acid residues 2903 and 3451 and contained all known viral RNA polymerase motifs A–E (Figure S4). We also found other motifs of orthonairovirus RdRp in the L segment of BJNV, including the zinc finger, leucine zipper, and ovarian tumour motifs (Figures S5–S7) [21, 22].

The S segment contained a 1659-nt ORF encoding a putative 552-aa nucleocapsid protein (N protein), which is associated with viral RNA synthesis and modulation of host immune responses in bunyaviruses [23, 24]. We also found a conserved domain at the C-terminal amino acid residues of the protein between BJNV and other nairo-like viruses or nairoviruses that may be essential for the important functions of the N protein (Figure S8) [25].

### Epidemiological investigation

**Figure 3.**
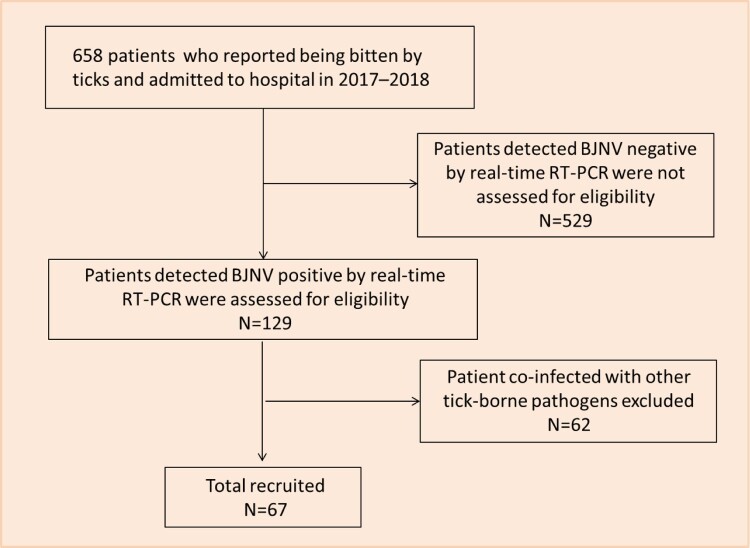
Flow diagram of recruitment of BJNV-positive patients.

During 2017–2018, a total of 658 patients who were bitten by ticks and admitted to hospital were collected and detected for BJNV and other tick-borne pathogens. We identified 129 patients with BJNV infection with complete medical records using real-time RT–PCR, in which 67 were identified only infection with BJNV, and the other 62 were co-infected with other tick-borne pathogens and excluded in the study (Figures 3, Figures S9 and S10). Among these patients, 43 patients were found in 2017, and 24 in 2018; 49 were from Inner Mongolia, and 18 from Heilongjiang province. Most BJNV infections (44 patients) occurred from May through July (Table S7). Of the 67 patients, 47 (70%) were men, all were farmers or forestry workers who lived in hilly or wooded areas and worked in fields, and 67 (100%) had a history of tick bites before the illness onset. The median age of patients was 48 years (interquartile range 41–53 years, range 28–85 years) (Table S7). The median incubation period was 7 days (interquartile range 3–12 days, range 2–42 days). The comorbidities included chronic cardiovascular disease (7.5%, 5/67), uncomplicated diabetes (6.0%, 4/67), and non-asthmatic chronic pulmonary disease (1.5%, 1/67); 89.5% (60/67) patients had no reported comorbidity.

**Figure 4.**
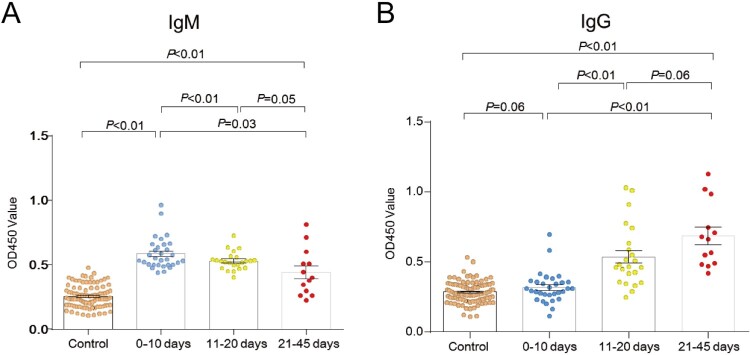
Virus-specific antibodies in patients detected by ELISA. OD_450_ values for IgM (A) and IgG (B) of serum samples from each patient are shown. The mean and standard error of the mean (SEM) are indicated for each group.

We evaluated the virus-specific antibody responses in infected patients by with an indirect ELISA using recombinant nucleoprotein (Figure S11), showing an IgM positive rate of 62.7% (42/67) and an IgG positive rate of 29.9% (20/67) (Table S8). We further classified all serum specimens into three groups based on the sampling time, showing a higher IgM titer in the 1–10 days group, with a downward trend in the 11–20 days and 21–45 days groups (Figure 4A). An upward trend of the average IgG titer was seen during the acute phase of infection (Figure 4B). We neither detected viral RNA nor specific antibodies against BJNV in the serum specimens from healthy individuals living in the same regions.

It is important to note that BJNV RNA was detected by a nested RT-PCR in multiple tick species, including *Ixodes persulcatus* (2.0%), *Ixodes crenulatus* (2.1%), *Demacentor silvarum* (2.1%), *Demacentor nuttalli* (0.4%), *Haemaphyslis concinna* (1.1%), and *Haemaphysalis longicornis* (0.4%) collected from hilly and wooded regions, with a prevalence ranging from 0.4–2.1% in different tick species (Table S9). A high prevalence of BJNV infection was found in ticks in Inner Mongolia (4.3%) and Jilin (3.3%) as comparison with that in Heilongjiang (1.0%). In addition, BJNV RNA was detected in serum samples that were collected from sheep and cattle in Inner Mongolia, revealing a high prevalence of 54.6% (118/216) in sheep and 37.7% (86/228) in cattle.

We further obtained partial viral RNA sequences from ticks and domesticated animals by RT-PCR assays, and phylogenetic analysis revealed that they were closely related to those present in human patients (Table S10 and Figure S12), suggesting that they may function the potential vectors and animal reservoirs for this emerging virus, respectively.

### Clinical features

The most common symptoms of the 67 BJNV-infected patients were fever (67 patients) and headache (66 patients). Other clinical findings included depression (42 patients), coma (42 patients), fatigue (36 patients), myalgia or arthralgia (30 patients), poor appetite (24 patients), and skin rashes or petechiae (21 patients). The clinical symptoms, including cough, chills, chest and/or abdominal pain and tightness, vomiting, lymphadenopathy, diarrhea, nausea, and itching were also observed in some of the patients (Table 1). Two (3%) patients became critically ill, and were admitted to an intensive care unit due to dyspnea; one died (Figure S13).

**Table 1.** Clinical characteristic of 67 patients with BJNV infection.

Clinical symptoms^a^	Patients with symptoms (%, *n* = 67)
Fever	67 (100)
Headache	66 (99)
Depression	42 (63)
Coma	42 (63)
Fatigue	36 (54)
Myalgia or arthralgia	30 (45)
Poor appetite	24 (36)
Skin rash or petechiae	21 (31)
Cough	15 (22)
Chills	12 (18)
Chest tightness	12 (18)
Vomiting	3 (5)
Lymphadenopathy	3 (5)
Abdominal pain or tenderness	3 (5)
Diarrhea	3 (5)
Skin itching	3 (5)
Nausea	2 (3)
dyspnea	2 (3)

^a^ Shown are prospectively collected clinical characteristics of patients with laboratory-confirmed BJNV infection.

Laboratory testing revealed an elevated level of high sensitivity C-reactive protein in 74.4% patients. Multiple organ dysfunction was seen, with the most common organ damage of liver, followed by heart and kidney. Overall, 31% (20/65) of patient had increased level of alanine aminotransferase, 39% (25/65) had increased aspartate aminotransferase, 41% (7/17) had increased lactate dehydrogenase, 15% (3/20) had increased creatine kinase, 8% (5/63) had increased creatinine and 6% (4/63%) had increased serum creatinine. The blood coagulation disorders were also observed in some patients, as shown by elevated levels of activated partial thromboplastin time (12%, 7/60) and D-dimer (33%, 3/9) (Table S11).

Most patients were treated with a combination of benzylpenicillin sodium and ribavirin. Benzylpenicillin sodium was administered 4 million units per day intramuscularly, and ribavirin was 0.5 g per day intravenously. The symptoms usually disappeared after treatment for 7–14 days. For the critically ill patients, mechanical ventilation was needed immediately when respiratory failure developed.

### Animal infection

**Figure 5.**
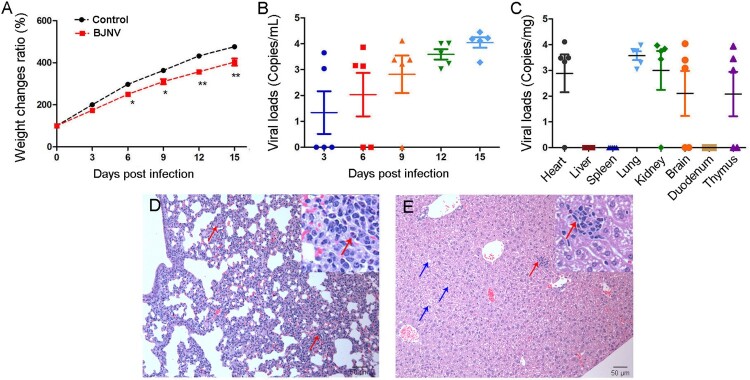
Animal infection with BJNV infection. Wild-type C57BL/6 suckling mice aged 1 day were inoculated with BJNV by the intracerebral route. The animals showed decreased body weight (A, *n* = 5), and viremia (B, *n* = 5) and viral loads in the tissues (C, *n* = 5) were detected by real-time RT-PCR. Viral loads were presented by common logarithm. Pathological changes of the lung (D) and hepatic (E) tissues were observed. Thickened alveoli septum and infiltrated inflammatory cells (Red arrow, ×100) in lung. Swelling (Blue arrow, ×100) and infiltrated inflammatory cells (Red arrow, ×100) in liver. Data are expressed as the mean ± SEM. * *P *< 0.05; ** *P *< 0.01.

Wild-type C57BL/6 suckling mice were inoculated intracerebrally with BJNV strain YKS44. The infected animals showed significant weight loss at 6, 9, 12, and 15 days post-infection (dpi) as compared to the control group (Figure 5A). Viremia began on the third day of infection and lasted until the end of the experiment (15 dpi) (Figure 5B). Moreover, virus could be found in multiple organs, such as heart, lung, kidney, thymus, and brain at 15 dpi (Figure 5C). Histopathological analysis showed thickened alveoli septum and infiltrated inflammatory cells in the lung tissues and hepatocyte swelling and infiltrated inflammatory cells in the liver tissues (Figure 5D, 5E)). BJNV could be isolated from the blood samples of infected mice at 15 dpi, and cause similar clinical manifestations in suckling mice as described above (data not shown).

## Discussion

The present study provided virological, epidemiological, and experimental results of the newly discovered nairo-like virus BJNV, showing that it may be the etiological agent responsible for the febrile illness in northeastern China. BJNV RNA was detected in 67 patients who had the clinical symptoms of fever and headache, while absent in healthy population. The virus has been isolated from the patients, and can infect several human cell lines. The isolated virus could cause disease in suckling mice that produce growth retardation, viremia, and histopathological changes. Re-isolated virus from the infected suckling mice could also induce viremia and histopathological changes in suckling mice.

Interestingly, BJNV belongs to a cluster of nairo-like viruses that are genetically closely related to nairoviruses, but seem to be different from the typical nairovirus species, as they are all apparently missing the medium M segment that codes for the viral glycoproteins. More recently, another virus very closely related to BJNV, Gakugsa tick virus, has been described in Russia. This nairo-like cluster consists of possible two other distinct viruses, including South Bay virus found in the United States of America and Grotenhout virus found in Belgium [4, 6]. Recently, two viruses, including Pustyn virus found in Russia and Norway nairovirus 1 found in Norway, are very closely related viruses to Grotenhout virus, suggesting that Grotenhout virus has an extended geographical spread. BJNV RNA has been detected in multiple tick species, such as *I. persulcatus*, *I. crenulatus*, *D. silvarum*, *D nuttalli*, *H. concinna*, *H. longicornis*, and domestic animals (sheep and cattle) in northeastern China, while Gakugsa tick virus has been found in *I. persulcatus* in Russia. Therefore, these ticks may be candidate vectors of BJNV, and the virus may also have a wide geographical distribution.

Our study has limitations. Firstly, we failed to obtain the potential medium segment of BJNV using meta-transcriptomics analysis (Supplementary material), as it usually encodes the glycoproteins in the typical nairoviruses. It is possible that the glycoprotein gene of these viruses may be highly divergent, or even non-homologous sequences. Surprisingly, the same phenomenon is also present in some newly discovered species in both phleboviruses (e.g. Tacheng tick virus 1) and chuviruses (e.g. Xīnzhōu nematode virus 5) [26]. However, the biological significance and cause of the incomplete viral genomes require further investigation. Secondly, a few BJNV-infected patients were found in the present study, therefore, an active surveillance should continue to find more patients and to confirm the clinical characteristics of this emerging disease different from other virus such as SFTSV, TBEV or ALSV. Thirdly, only serum specimens were obtained from the acute phase of the illness, and the serum specimens at the convalescent phases should also be collected to determine dynamic changes of virus-specific antibodies. Finally, the present study has not completely fulfilled the Koch’s postulates of identification of a novel etiology associated with an emerging disease, and an animal infection model should further be established to reflect the clinical characteristics of BJNV-infected patients [27].

Ticks can transmit a large number of pathogens to humans, in which most infections may show similar clinical characteristics [2, 28]. The clinical feature of BJNV infection is difficult to differentiate from SFTSV, TBEV, Babesia, Anaplasma, or Lyme disease spirochetes infection. Fevers and headache are the same clinical manifestation of TBEV, SFTSV, ALSV, SGLV, anaplasmosis and BJNV infection. Central nervous system symptoms often occur in meningitis, encephalitis and TBEV infection. Patients with SFTSV or anaplasmosis are manifested as leukopenia and thrombocytopenia, and *Babesia* infection presents with pyretotyposis, splenomegaly, jaundice, or hemolysis etc. Erythema migrans is the early clinical hallmark of Lyme disease, and some patients in advanced stages shows nerve and cardiac abnormalities, musculoskeletal symptoms, or periodic joint damage. However, BJNV infection is not always easy to differentiate from ALSV and SGLV resulting from its similar clinical feature (fevers and headache) in early infecting stages. For now, molecular biotechnology is the best methods to differentiate these viruses (BJNV, ALSV and SGLV) infection. Therefore, more active monitoring patients with BJNV infection to confirm the typical clinical characteristics is urgent and necessary.

Importantly, migratory birds can act as long-distance disseminators of ticks and the tick-borne pathogens, suggesting that BJNV may have potential global public health implications [29, 30]. BJNV is only one of the emerging viruses that have been identified in China, in addition to SFTSV, Alongshan virus, Songling virus, and Tacheng tick virus 1, highlighting the importance of disease surveillance in the discovery of emerging infectious diseases. In summary, our findings suggest that BJNV may be the cause of a previously unknown febrile disease, and its public health significance necessitates further research in a large geographical region.

## Supplementary Material

Supplemental_material-R1.doc

## Data and materials availability

All data associated with this study are available within the article and its supplementary information. The viral sequences involved in the study have been deposited in GenBank Nucleotide database.
