# Supplementary material for: A new nairo-like virus associated with human febrile illness in China
Source: Emerg Microbes Infect. 2021 Jun 17;10(1):1200–8. doi: 10.1080/22221751.2021.1936197 (PMC8212832; doi:10.1080/22221751.2021.1936197)
Supplement: Supplemental_material-R1.doc [file TEMI_A_1936197_SM6551.doc]

**Supplementary material**

**Methods**

**Sample processing and sequencing**

Total RNAs from collected specimens were extracted by using RNeasy Plus minikit (Qiagen) according to the manufacturer’s instructions. The host ribosomal RNA (rRNA) was removed using the Ribo-Zero-Gold (Epidemiology) kit (Illumina Inc., USA), and sequencing library construction was performed by using a TruSeq total RNA library preparation kit (Illumina) following the manufacturer’s instructions. Paired-end (150-bp) sequencing was then performed on the Illumina Hiseq Xten platform.

**Sequence assembly and RNA virus discovery**

Sequencing reads were demultiplexed and trimmed for quality, and then assembled *de novo* using the Megahit (1). The assembled contigs were first compared to the database of all reference RNA virus proteins downloaded from National Center for Biotechnology Information (NCBI) by using the BLASTX with an expect value cutoff of 1E-3, and potential viral contigs were further compared against the entire nonredundant protein (nr) database to confirmation. The quality-filtered virus contigs with unassembled overlaps were then merged by using the SeqMan program implemented in the Lasergene software package. To confirm the assembly results, sequencing reads were re-mapped to the target contigs with Bowtie2 (2) and inspected by using Integrated Genomics Viewer (IGV) (3) for any assembly errors.

**Sequence confirmation and complete viral genome determination**

Viral contigs were confirmed through overlapping RT-PCR using the primers designed base on the assembled sequences, the gaps were filled by RT-PCR and Sanger sequencing, and the 5' and 3' termini of viral RNA segments were determined with a RACE kit (Takara).

**Results**

Through the unbiased high-throughput RNA sequencing approach, a total of 6.39 G clean bases were remained after passed default quality control (QC) filters on the Illumina platform and removed the adapter sequences, resulting in 21,312,369 paired-end sequencing clean reads, and generated a total of 310,556 contigs, which varying from 353 to 14825 nt in length. After compared to the reference RNA virus protein and nr databases, 89 contigs were annotated as viral contigs, and the length range from 424 to 14825 nt. Aomg all viral contigs, two contigs that 14825 nt and 2710 nt in length were annotated as Beiji nairovirus RNA-dependent RNA polymerase (AXQ59276.1) and Pustyn virus nucleocapsid protein (ANJ43352.1), with 97.9% and 86.9% animo acid identity, respectively. No prospective glycoprotein candidates belonging to the family *Nairoviridae* were found by blast searches. Using these two contigs as the reference sequences, 11,920 and 7,493 reads were remapped to the corresponding reference sequence at a mean depth of 120× and 414×, with pairwise identity of 99.5% and 99.4%, respectively.

To search for the potential M segment of BJNV, extensive BLAST (basic local alignment search tool) and HHblits (iterative homology detection by representing both query and database sequences by profile hidden Markov models) screening of the nr database were performed. No *Nairoviridae* glycoproteins were detected with the use of either algorithm. Then, we screened all assembled high-throughput sequencing contigs with abundance range from 10 to 1000, since that of L and S segment was 100 and 423, respectively. Simultaneously, we excluded the contigs that less than 1000 nt in length, since the near complete L and S segment could be assembled from high-throughput sequencing reads, and the length of complete glycoprotein gene of other reference Orthonairovirus range from 4024 nt to 5366 nt. No contigs could simultaneously fullfill the following criterion: (i) an N-terminal signal domain, (ii) a C-terminal transmembrane domain, (iii) putative glycosylation sites, and (iv) comprise a single, continuous ORF and encodes a single protein, and then cleavage into two proteins.

**Refenerces**

1. Li, D., Liu, C-M., Luo, R., Sadakane, K., and Lam, T-W., (2015) MEGAHIT: An ultra-fast single-node solution for large and complex metagenomics assembly via succinct de Bruijn graph. Bioinformatics, doi: 10.1093/bioinformatics/btv033 [PMID: 25609793].

2. Langmead B, Salzberg SL. 2012. Fast gapped-read alignment with Bowtie 2. Nat Methods 9:357–359.

3. Thorvaldsdottir H, Robinson JT, Mesirov JP. 2013. Integrative Genomics Viewer (IGV): high-performance genomics data visualization and exploration. Brief Bioinform 14:178 –192.


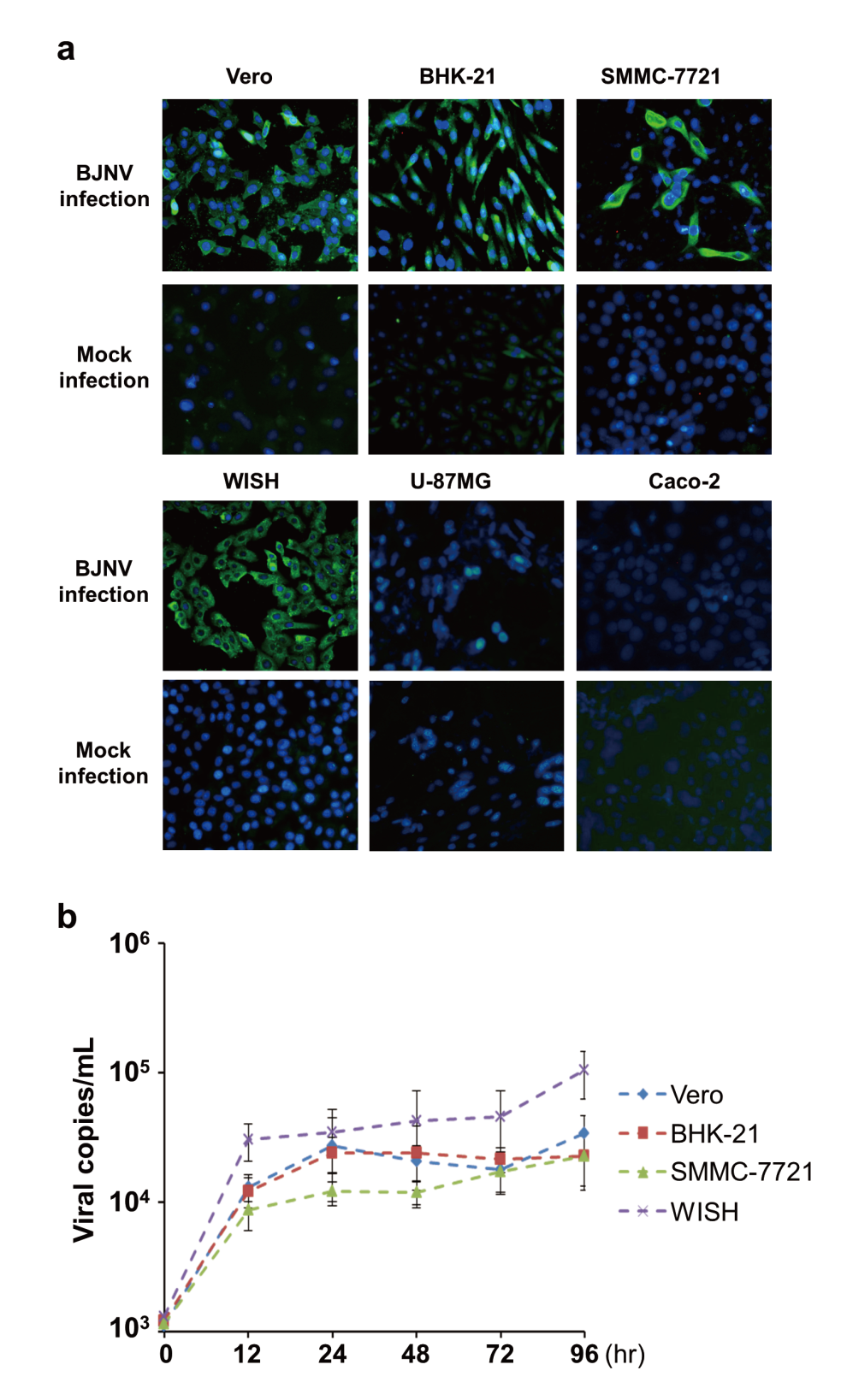


**Fig. S1.** **Infection of BJNV in different cell lines. a**, Mock or BJNV-infected cells at a MOI of 5 were stained with BJNV positive human or sheep serum samples (red) and DAPI (blue). Non-infected cells were used as negative controls (mock infection). The experiment was conducted independently three times with similar results. **b**, Replication kinetics of BJNV in infected cells. The cells were infected with BJNV at a MOI of 5 in triplicate. The supernatants of infected cells were collected at times indicated and virus was determined by real-time RT-PCR; results from the three technical replicates were averaged and error bars indicate standard deviation across biological replicates. WISH, human amniotic cells; SMMC-7721, human hepatocarcinoma cells.

**
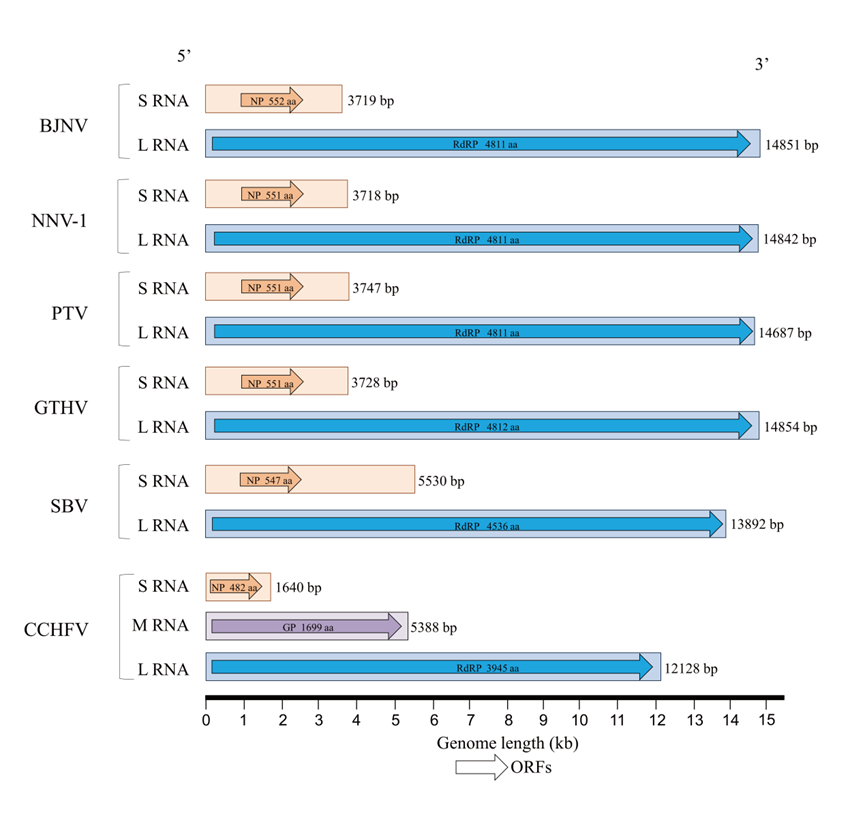
**

**Fig. S2. Schematic genome organization and putative proteomic maps of BJNV and the related viruses.** The locations of the NP, GP and RdRP ORFs are shown with orange, purple, and blue arrows separately. Positions and length of ORFs and segments are shown in the figure. Abbreviations and GenBank accession numbers of the virus are as follows: BJNV, Beiji nairovirus (L: MN122079, S: MN122080); NNV-1, Norway nairovirus 1 (L, MF141040; S, MF141041); PTV, Pustyn virus (L, KT007142; S, KT007143); GTHV, Grotenhout virus (L, KY700684; S, KY700683); SBV, South Bay virus (L, KM048320; S, KM048321); CCHFV, Crimean-Congo hemorrhagic fever virus (L, KY484046; M, KY484047; S, KY484048).


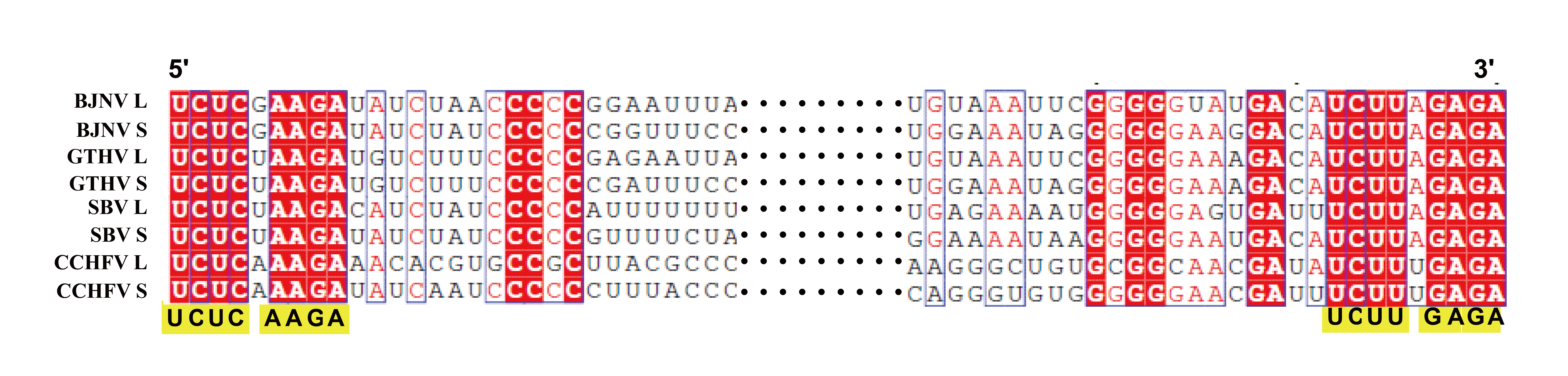


**Fig. S3.** **The terminal reverse complementary sequences of BJNV and related viruses.** The 5'- and 3'- termini of BJNV, GTHV, SBV and CCHFV were marked and highlighted in yellow under the aligned sequence. The 5'-terminal sequence typically consists of UCUCG/U/AAAGA, and 3'-terminal sequence consists of reverse compliment sequence with AGAGU/AUUCU. BJNV, Beiji nairovirus; NNV-1, Norway nairovirus 1; PTV, Pustyn virus; GTHV, Grotenhout virus; SBV, South Bay virus; CCHFV, Crimean-Congo hemorrhagic fever virus.


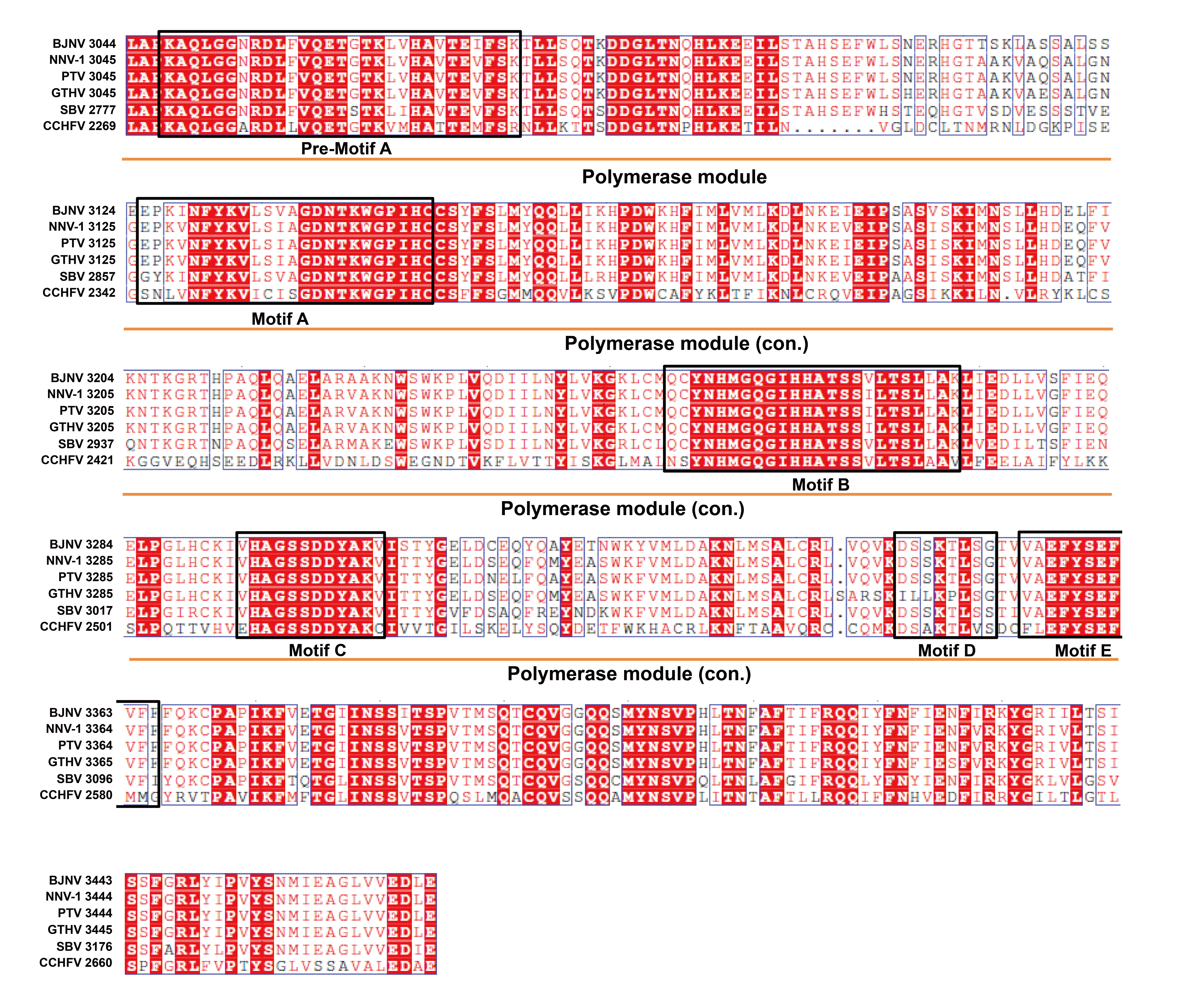


**Fig. S4. Amino acid sequence alignments of RNA-dependent RNA polymerase motifs in nairoviruses and nairo-like viruses.** Motifs from pre-A through E were signed with black boxes. BJNV, Beiji nairovirus; NNV-1, Norway nairovirus 1; PTV, Pustyn virus; GTHV, Grotenhout virus; SBV, South Bay virus; CCHFV, Crimean-Congo hemorrhagic fever virus.


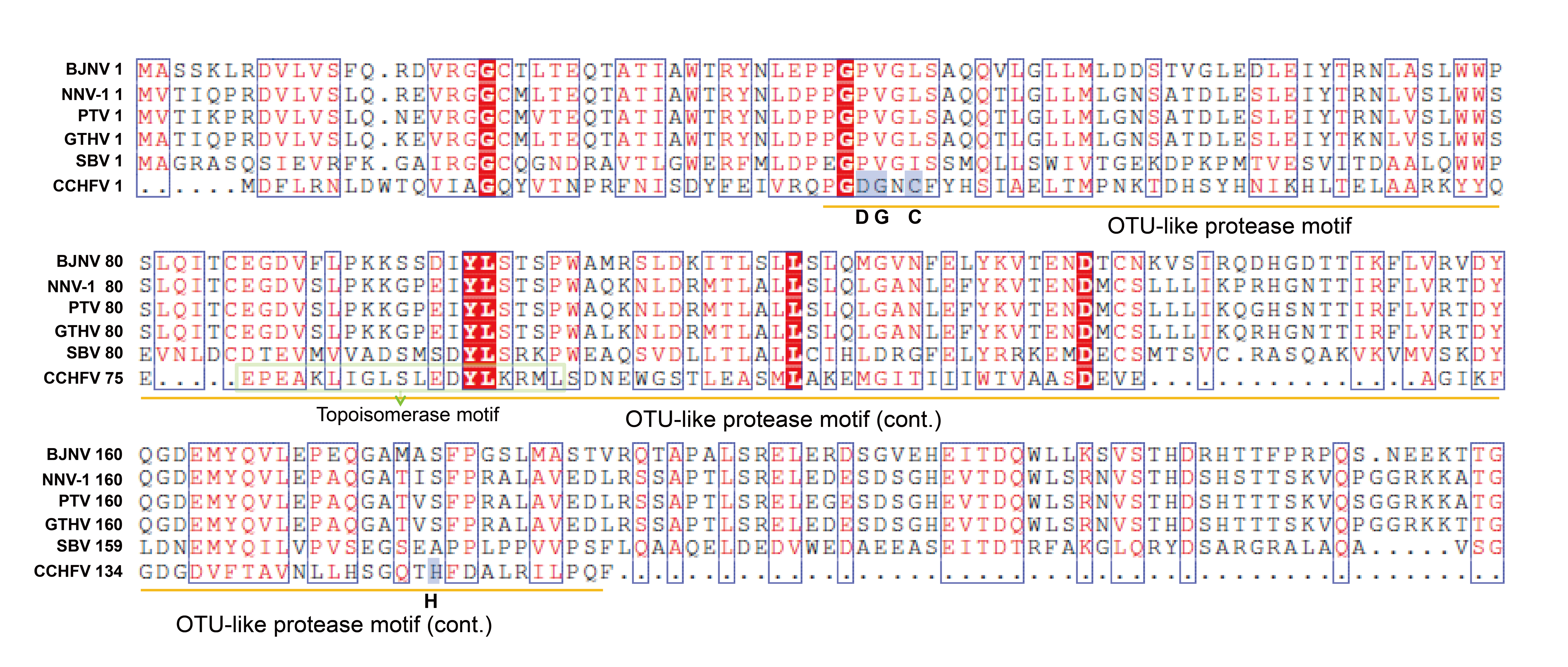


**Fig. S5. Alignment of the N-terminal of the L protiens from nairovirus and nairo-like viruses**. Ovarian tumor domain (OTU)-like cysteine proteases motif of CCHFV was lined with orange, and the conserved catalytic residues (D37, G38, C40, and H151) in CCHFV were marked and highlighted in blue. The N-terminal topoisomerase motif of CCHFV was signed with green box. BJNV, Beiji nairovirus; NNV-1, Norway nairovirus 1; PTV, Pustyn virus; GTHV, Grotenhout virus; SBV, South Bay virus; CCHFV, Crimean-Congo hemorrhagic fever virus.


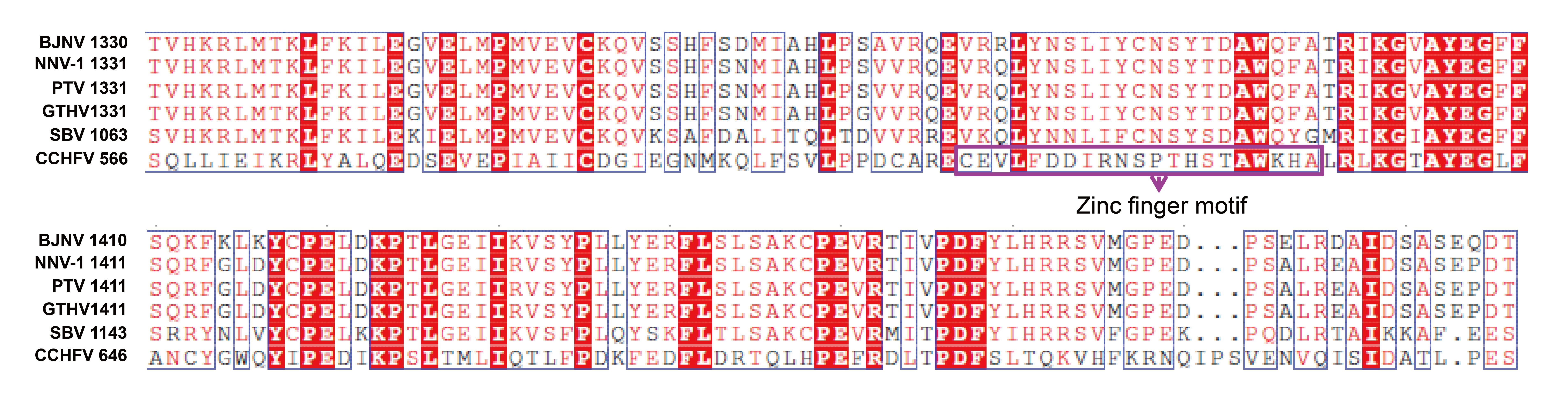


**Fig. S6. Alignment of the zinc finger motif of L proteins from nairoviruses and nairo-like viruses.** The zinc finger motif of CCHFV was signed with purple box. BJNV, Beiji nairovirus; NNV-1, Norway nairovirus 1; PTV, Pustyn virus; GTHV, Grotenhout virus; SBV, South Bay virus; CCHFV, Crimean-Congo hemorrhagic fever virus.


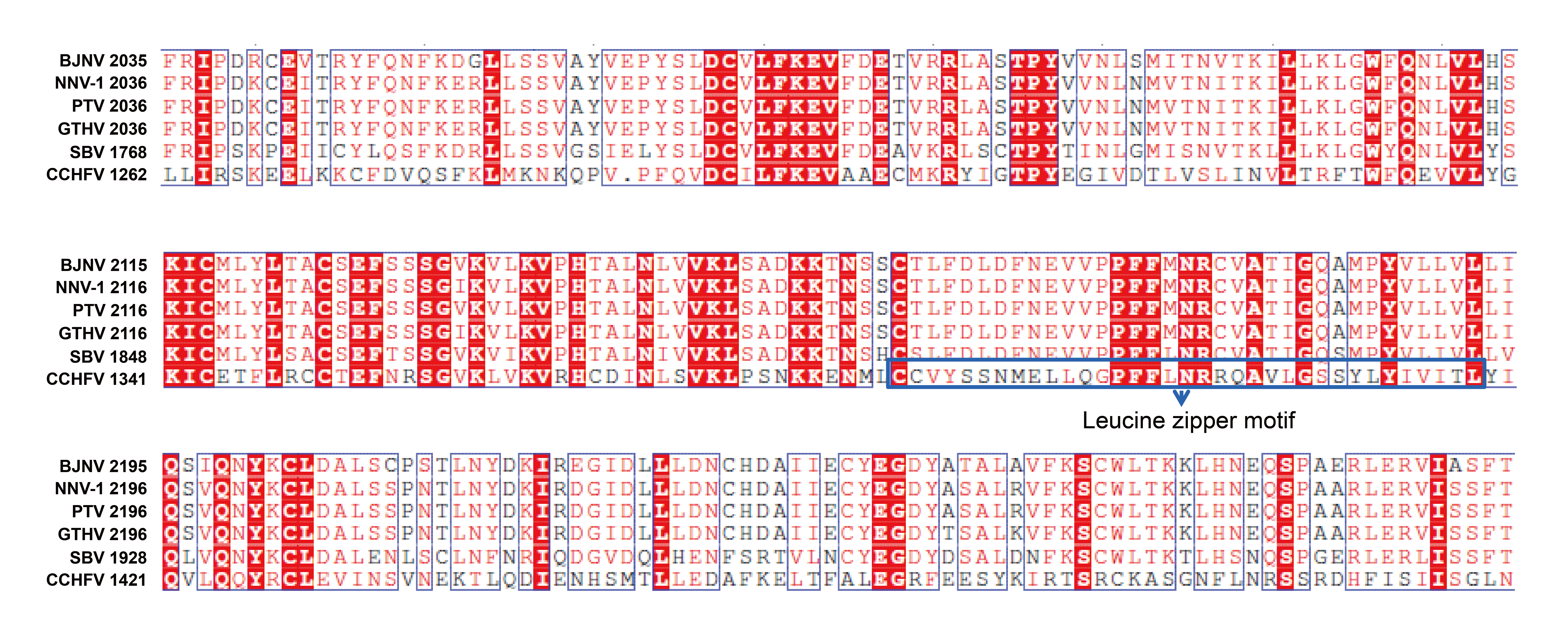


**Fig. S7. Alignment of the leucine zipper motif of L proteins from nairoviruses and nairo-like viruses.** The leucine zipper motif of CCHFV was signed with blue box. BJNV, Beiji nairovirus; NNV-1, Norway nairovirus 1; PTV, Pustyn virus; GTHV, Grotenhout virus; SBV, South Bay virus; CCHFV, Crimean-Congo hemorrhagic fever virus.


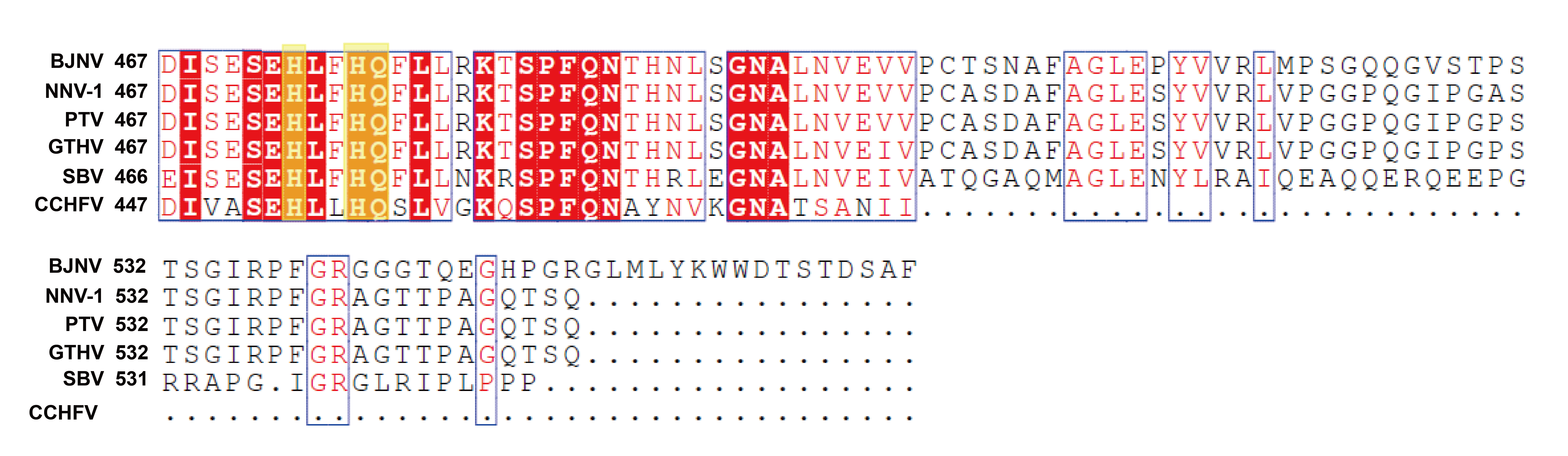


**Fig. S8. Alignment of the conserved C-terminal domain present in N proteins of nairoviruses** **and nairo-like viruses**. Residues implicated in N function are highlighted in yellow. The sequences were showed by ESPript 3.0 (http://espript.ibcp.fr/ESPript/cgi-bin/ESPript.cgi). Conserved residues are indicated with red backgrounds. The amino acid position numbers within the N and L proteins are displayed for each virus. BJNV, Beiji nairovirus; NNV-1, Norway nairovirus 1; PTV, Pustyn virus; GTHV, Grotenhout virus; SBV, South Bay virus; CCHFV, Crimean-Congo hemorrhagic fever virus.


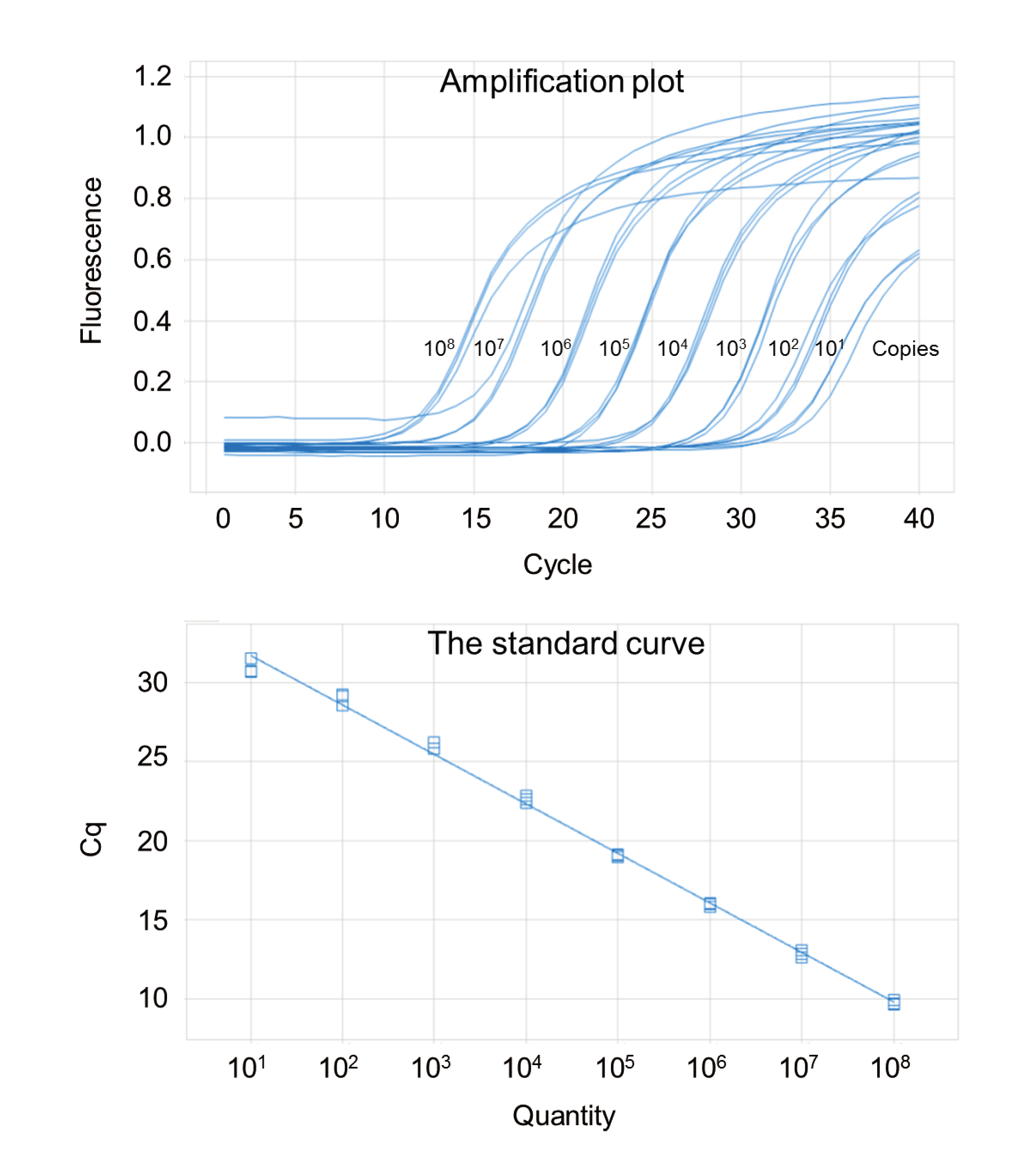


**Fig. S9.** Development of real-time RT-PCR for BJNV detection. Amplification plot of plasmids containing the SGLV nucleoprotein gene as template for real-time RT-PCR, which was serially diluted from 10^8^ to 10^1^ copies was used as template (top panel). The standard curve for the real-time RT-PCR is shown in lower panel. The used primers and are shown in the supplementary table S1, and the reaction conditions are shown in the Method.


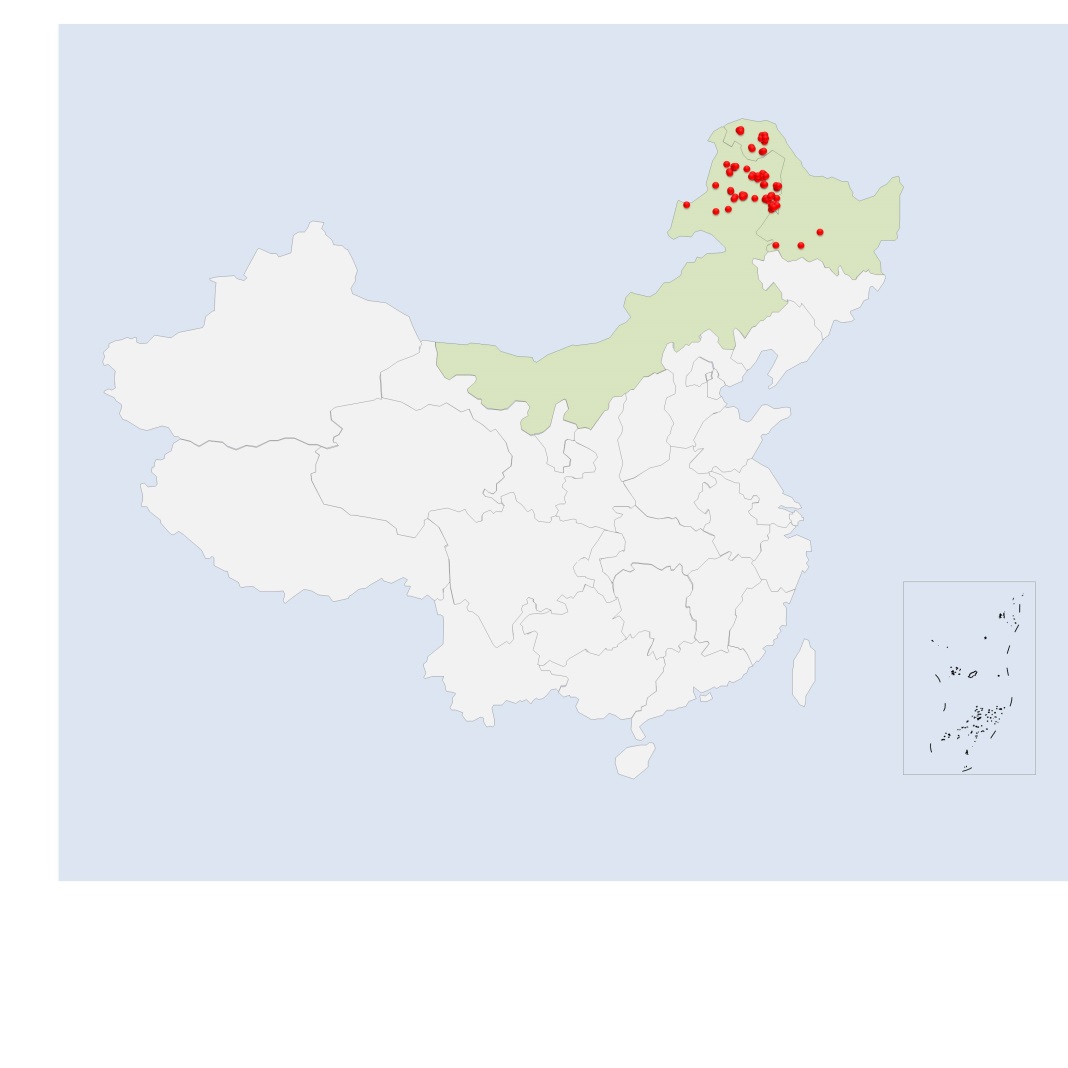


**Fig. S10. Geographic distribution of patients with BJNV in China.** Areas where surveillance of tickborne pathogens was carried out are shown in green. Red dots indicate the locations of the patients with laboratory-confirmed BJNV infection.


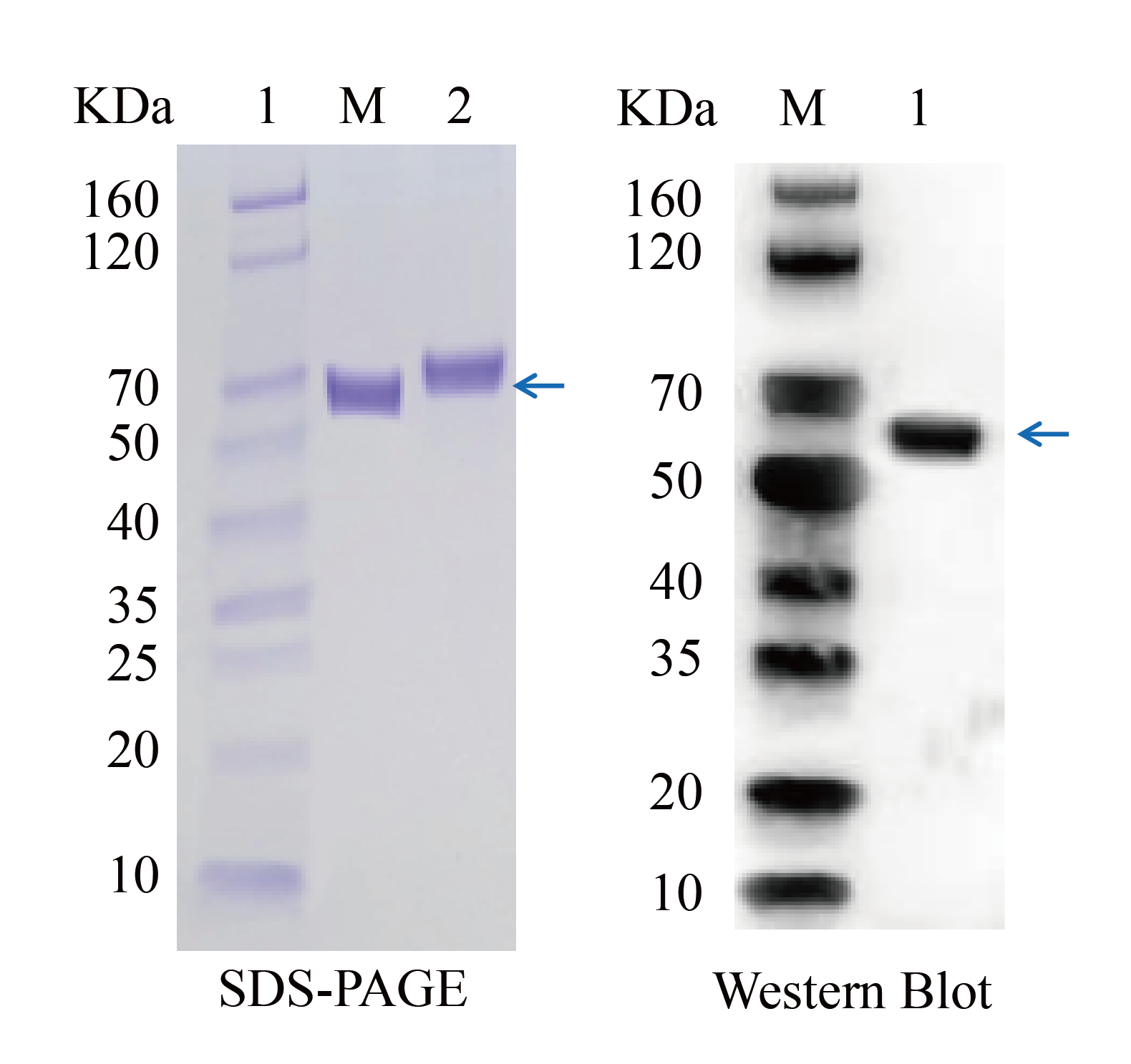


# Fig. S11. Recombinant nucleoprotein of BJNV used in ELISA assay. Sodium dodecyl sulphate-polyacrylamide gel electrophoresis (left panel) showed purity of his-tagged nucleoprotein of BJNV (lane 2) and albumin from bovine serum (lane 1). Western-blot analysis of recombinant nucleoprotein using anti-His monoclonal antibody (right panel). Lane M is protein molecular weight marker.


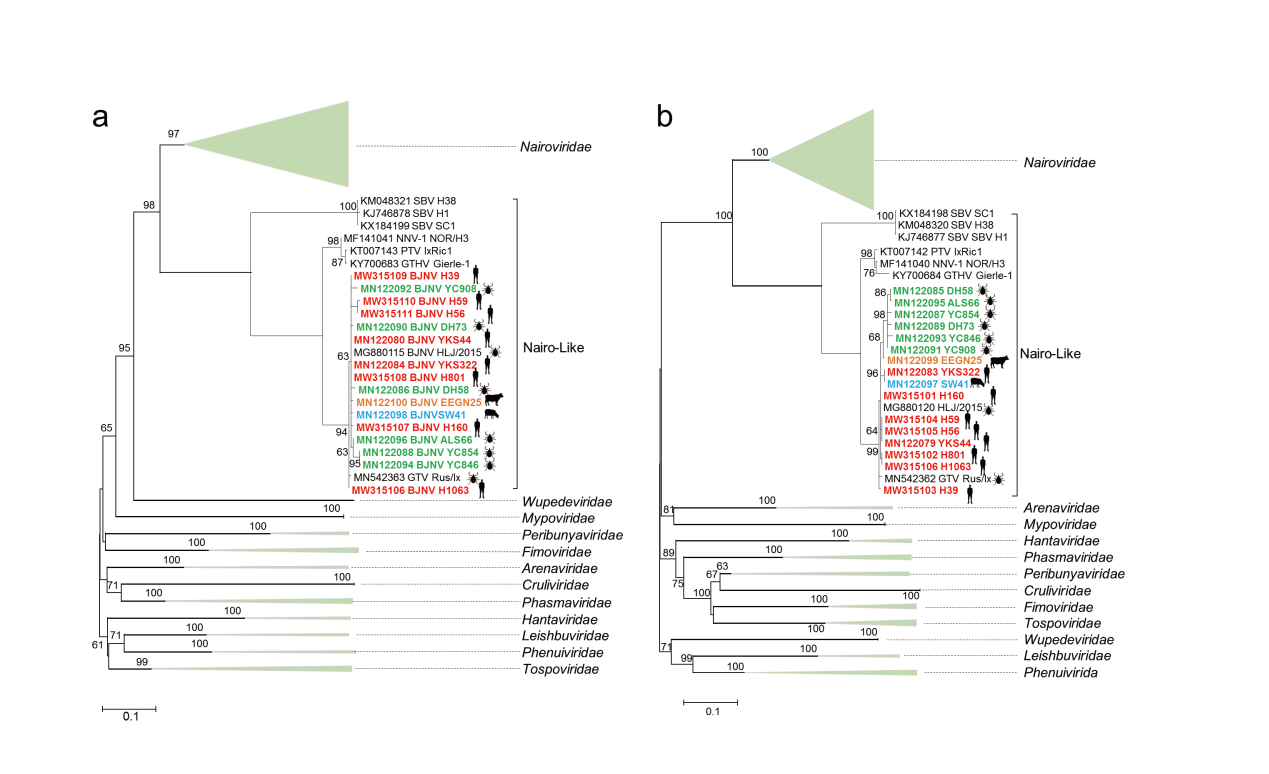


**Fig. S12.** **Phylogenetic relationship of BJNV from different origins.** a, The phylogenetic trees were generated by MEGA5 software from partial S segment. b, The phylogenetic trees were generated by MEGA5 software from partial L segment. The trees were analyzed by the Maximum likelihood method with the Jukes-Cantor model. Bootrap testing of 1000 replicates was performed, and the bootstrap values are indicated. The viruses in the in the order *Bunyavirales* used for phylogenetic analysis are shown in the Supplementary Table 3. Sequences are identified by their GenBank accession numbers, followed by the virus name and strain. The scale bars in each panel indicate 0.05 substitutions per site. BJNV, Beiji nairovirus; GTV, Gakugsa tick virus; NNV-1, Norway nairovirus 1; PTV, Pustyn virus; GTHV, Grotenhout virus; SBV, South Bay virus.


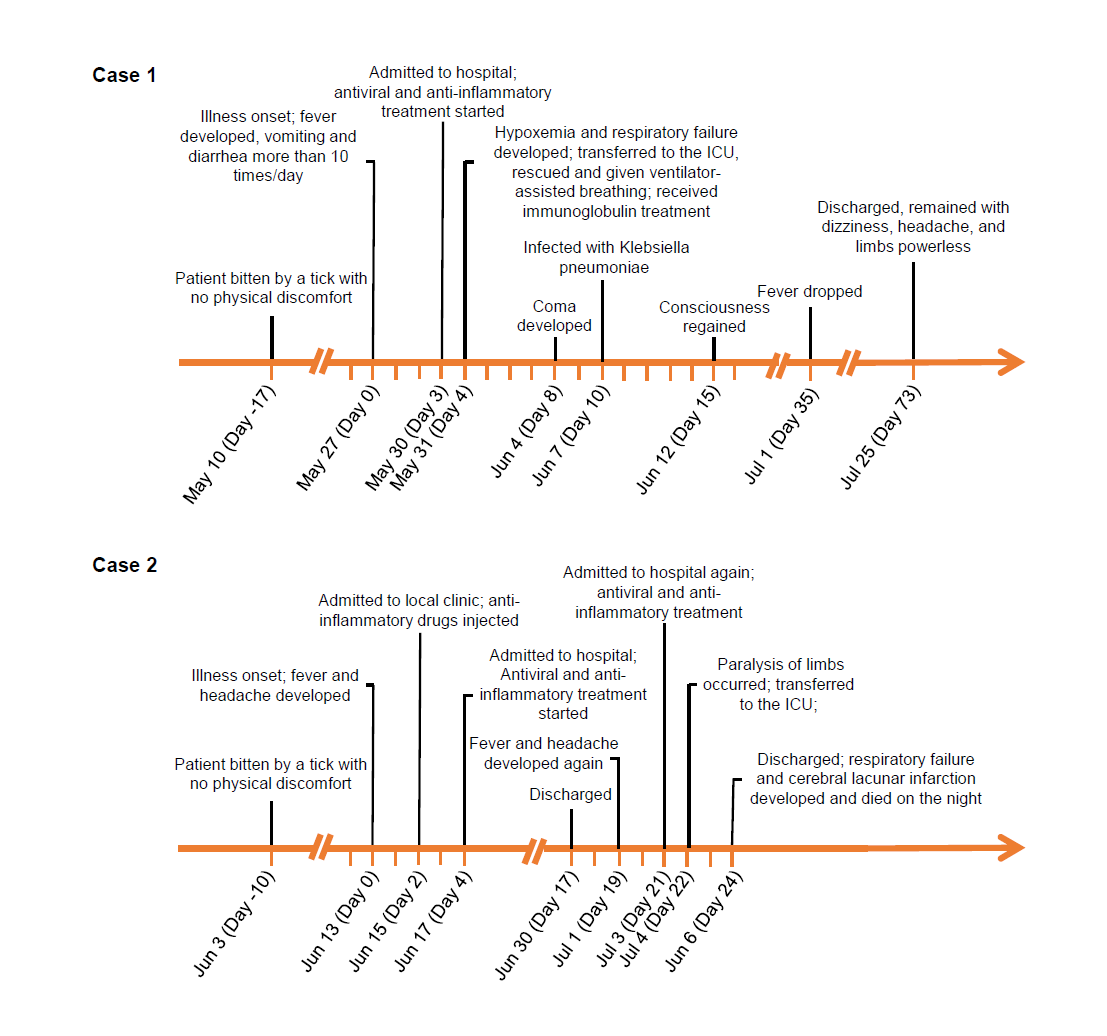


**Fig. S13. The clinical course of the two critically ill patients with BJNV infection in 2017.** Case 1 was a 51-year-old man without any comorbidity, and case 2 was an 85-year-old woman with a comorbidity of pulmonary tuberculosis.

**Table S1.** Primers used in the nested RT-PCR and real-time RT-PCR to detect BJNV.

| Prime ID^*^ | Primer sequences | PCR product (bp) |
| --- | --- | --- |
| P1 | Forward: 5'–TTG GTG ATA TTG ATG AAA GCA AGA GTG AG–3' | 431 |
| P2 | Revers: 5'–RTC AAA CAC YCG TAG TGG TCC AGG AAC–3' |
| P3 | Forward:5'–TTG GTG ATA TTG ATG AAA GCA AGA GTG AG–3' | 337 |
| P4 | Revers: 5'–GCW CGR TGT ATT ACW ATC TCT GGG AAC TT–3' |
| P5 | Forward: 5'–TCA CCA GTT CCT ACT ACG–3' | 90 |
| P6 | Revers: 5'–GGA CAA CCT CAA CAT TCA–3' |

^*^P1 and P2 were used for first-round PCR; P3 and P4 were used for the second-round PCR; P5 and P6 were used for real-time RT-PCR.

**Table S2.** Primer sequences used to amplify the complete genome of BJNV.

| **Genome segment** | **Forward (location) (5'→ 3')** | **Reverse (location) (5'→3 ')** |
| --- | --- | --- |
| **Large segment** | L-5’Race-RSP1: CTAATACGACTCACTATAGGGCAAGCAGTGGTATCAACGCAGA | L-5’Race-GSPs1: CTCAAAGTTCACTCCCATCTG |
|  | L-5’Race-RSP2: CTAATACGACTCACTATAGGGC | L-5’Race-GSPs2 CCATCTGTAGTGAAAGTAGGGC |
|  | LF426: YCGGAKCWYTRCARATHWCATGYGAAGG | L1723R2: CTGGGTTCTYTGATTCCTGCTTTA |
|  | LF1715: AGAACCCAGAGTTCTACAAGGACTTC | LR2065: GTCTCATATCACCYTGATACAGC |
|  | LF2043: GCTGTATCARGGTGATATGAGAC | LR3194: TCTGCATTACAGAKCATRCTYTCAGGAAC |
|  | LF3166: GTYCCTGARAGYATGMTCTGTAATGCAGA | LR3885: CATAAGGTCCATGCTAATAGACTG |
|  | LF3790: ATAAAAGARTACTTTGARGATTGGAGTG | LR4745: CCHGATGTRAGTTCCATTGGGTCTTCC |
|  | LF4723: GGAAGACCCAATGGAACTCAC | LR5822: CTGTCAACCTCTTCCATGTGTGC |
|  | LF5677: GCTGTGCCAAACATATACATAACTTACC | LR7553: CTTAGTTCTTGTAATGCCTGCTTAACCTGC |
|  | LF7613: TTGGTGATATTGATGAAAGCAAGAGTGAG | LR7949: GCWCGRTGTATTACWATCTCTGGGAACTT |
|  | LF7917: GAGAAAGTTCCCAGAGTATGTAATA  LF10845: DGCTGAYAAAGTCCCMAGACAATTAYTRG | LR12303: ACCTCTCTGAGTAAGTTGAATAGTC |
|  |  | LR12624: ATTTARWGCAAACATAGMAAGGTAA |
|  | L-3’Race-RSPs1: GTTGCACTCCTTCACATGTTCC | L-3’Race-GSP1: CTAATACGACTCACTATAGGGCAAGCAGTGGTATCAACGCAGA |
|  | L-3’Race-RSPs2: GTCCTACTCATTGGGTGTTCC | L-3’Race-GSP2: CTAATACGACTCACTATAGGGC |
| **Small segment** | LF14156: TGKCCAGCTTCTTRAGTGTCTG | LR14792: CAAAACTGTTAGGAGAAATGATGACT |
|  | SF2411: ATGCATTTGCTGGCCTAGAG | SR3710R: TGTCATACCCCCGAATTTACAAAG |
|  | S-5’Race-RSP1: CTAATACGACTCACTATAGGGCAAGCAGTGGTATCAACGCAGA | S-5’Race-GSPs1: CCCTCCTCAGTCCTTCATATAG |
|  | S-5’Race-RSP2: CTAATACGACTCACTATAGGGC | S-5’Race-GSPs2: GTAGGTTCGGTCATTCACATCC |
|  | S-3’Race-RSPs3: GCATGGGTGAGTAGGTTCC | S-3’Race-GSP1: CTAATACGACTCACTATAGGGCAAGCAGTGGTATCAACGCAGA |
|  | S-3’Race-RSPs4: CTCCACACCAAGCACCTCAG | S-3’Race-GSP2: CTAATACGACTCACTATAGGGC |

**Table S3**. Sequences of contigs of BJNV by metagenomic analysis.

| **Segment** | **Contigs** | **Sequence (5′→3′)** |
| --- | --- | --- |
| **S** | 1 | ATGCCTGTTGCCTTGCCCAAGTTTGCAACGAAGGATGATCTTGACAGGTGGTTTAATGCACTTACCCGGATGTGAATGACCGAACCTACTCCCCATCATATTTCGATGGGCTTCCTGATCTAGACTTGTATCTCGAGGAGATTCGCAGAGCAAACAGCAGGAGCTCTGCAGATATCATGGGTGAGTAGGTTCCTATATGAAGGACTGAGGAGGGCCCCGCCTCTAAGGGAGCAAGCATGGCTCATGAACGCAACTTTTTTTGAAAAGGCTATGAGGCACTTGACCAGTGAGGCAGGCAAAGATGGCACTCCAGTGAAAAAGCTTCTGACCATTTACCCAGCCTACCTTGAAGCAGGGTCAAAGTGCACAGACCCAGAGGTCATCAAGACCTACCAACTAGCATGCAGGAAGTTCCGAACTGCTATGGACCTGAGTGAACCCAATTCCCTGACAGGGATACTGTATGGGTCAGTTGCAACGAAGGTTGAAGT |
|  | 2 | GATGTGAATGACCGAACCTACTCCCCATCATATTTCGATGGGCTTCCTGATCTAGACTTGTATCTCGAGGAGATTGACAAACAAACGACAAGAGCAGGGAAAGATGCATGGGTGAGTAGGTTCCTATATGAAGGACTGAGGAGGGCCCCGCCTCTAAGGGAGCARGCATGGCTCATGAACGCAACTTTTTTTGAAAAGGCTATGAGACACTTAACCAGTGAGGCAGGCAAAGATGGCACTCCAGTGAAAAAGCTTCTGACCATTTACCCAGCCTACCTTGAAGCAGGGTCAAAGTGCACAGACCCAGAGGTCATCAAGACCTACCAACTAGCATGCAGGAAGTTCCGAACTGCTATGGACCTGAGTGAACCCAATTCCCTGACAGGGATACTGTATGGGTCAGTTGCAACGAAGGTTGAAGTCCCAGCCACCTTGATCTCAGTCTTCAGAGCTATGCTGCTGCGCATGAAACATCAGCTGGAGGGAAGGTTGGGTGTAGAAGGAAAAGAGAAAACTGATTCAACCAAGTTCCTGACACAGGTTAAGGC |
|  | 3 | ATGGACCTGAGTGAACCCAATTCCCTGACAGGGATACTGTATGGGTCAGTTGCAACGAAGGTTGAAGTCCCAGCCACCTTGATCTCAGTCTTCAGAGCTATGCTGCTGCGCATGAAACATCAGCTGGAGGGAAGGTTGGGTGTAGAAGGAAAAGAGAAAACTGATTCAACCAAGTTCCTGACACAGGTTAAGGCCTTTCTCAGATCTCTGGGAACCATCCTTCCTCCAGTCCCACTAACCGGAGCTCGCAGATATCAAAGGGTGGGCACCAAAGGAAGGAGCAGCAGAGCAAAGTGCCGAAAATGGAGCGTACCTGTTAACAA |
|  | 4 | GCACCAAAGGAAGGAGCAGCAGAGCAAAGTGCCGAAAATGGAGCGTACCTGTTAACAACTGCAATCATGCAGCTCCGG |
| **L** | 5 | CCTGGAAATTTACACCAGGAATCTCGCCAGCTTATGGTGGCCGGAGCTCTGCAGATATCATGCGAAGGGGATGTATTTCTCCCTAAGAAAAGCTCTGACATTTATCTGAGTACCTCTCCCTGGGCAATGAGAAGTCTGGACAAGATAACCTTGGCCCTACTTTCACTACAGATGGGAGTGAACTTTGAGCTTTATAAGGTTACTGAGAATGACACATGTAACAAGGTTTCCATCAGACAAGACCATGGCGACACCACCATAAAGTTCCTTGTGAGGGTAGACTACCAAGGTGATGAGATGTATCAGGTGTTAGAGCCTGAGCAAGGAGCCA |
|  | 6 | TGGAAGATACAGGTTGAGTAAGTGCCTGGAGCAAGAAAGGAAGAAAAGGTTGAAGAAGAAAGAACTAAAGCAGGAATCAGAGAACCCAGAGTTCTACAAGGACTTCATACCAGTGACTGGGCGCCCTGTGTTGCCATCCGGTGT |
|  | 7 | CTCCATGATGAAATTATCAATGATGGTAGTGCACATTATACTCAGTCTATTAGCATGGACCTTATGAAGTTAAGTGCTGTGGTTGATAAGTGCCTTGAGACTTTCAATGCACTTTACCCTAATGGAGACACCAGAGTTCCTGCCAAGGACAGTCAAGAAATCACATCTATAAGGATATCAGCTATGACTGCTATAAAAGAATACTTTGAGGATTGGAGTGCAGGACATAGCTATGTCTTAGTTAACCCAGCTATGCTAGAAGCTAGCATAAAGTGCTTGGCACAACATGCCAGTAGGATAGCCAGTTACCGTGAA |
|  | 8 | CACCTGCTACCTCTGATGACGAAGAAGTCACAATCGAACCTTCAATGGAAGACCCAATGGAACTCACATCTGGCCAGTCAAAGTACAAAATTGCTCCTGATGAGCCAAGTGGGACAAAGAAACAGTTTTCTCAGGCAACTCCGGAGCTCTGCAGATATCATTCGGTCAAAATGTGTTAGGGCAATATCCACCCGAGGCCAGCTTGGTAGGATAGTCAAAGACACAGTTGTTGTTGCTGAACACTCTGCAGATGATGAAAGTCCCATTGTGTGTAAGGTTTCACCTGCTTCATCACAAGAACGTGAGTTAATAATTTTTGAGGTTGGATTTGTTACAAATCCAGAACAAAAAATTGAAATTGACATGTCAAAATGGGGGAAAGCCATTAGAATTTTAGAAATGCTAAAGATTGGTACAACCCTGATAATTGCCACAGATGTATCTTCAAGATCAATTGATAAGTGGTGGATCTCACCATCAAGTGCAGGGCTGTTAAAAAGGAGTGTTGGAACCCTCTTCTTCTACCTTGTGAAACACACACCTCATGAGATTAAGGATCGAATAGTTAGTGGTTTATCTACATTAAAGCACAGTGTTAATAGAAAGGCAGGCTCAACTGTAAAGACCCCTGTAACTGTTGCTGATGTTAAGGAATACTTTGTTGAGGGGA |
|  | 9 | GGTCTATAGCAGTACTGGAAGAACTGGCAGACCGACACTTTAGTTGGGAAGCTCATGTAACAAAGATGGTAGATATTGTTAGGAGCCCTGATAGTAGTAGGAGGCAGGTTAAGCAGGCATTACAAGAACTAAGATTGTTAGTAGGAGCAGTGAAGTTGGATCAATTACCTTTAAGGAAAGATGATTCAGACTTTGGAGATATTGATGAAAGCAAGAGTGAATGTTCAAGGGGCTCCAGTTCTTTCAGAGCTTCATCAGTAGTTTCAAGTGTTAGAAGCTGGGGACGTGAC |
|  | 10 | GGGGTGCCCTGAGATTGTACAGGCAATGACGGAGTATGGCAAGAGAAAGTTCCCAGAGATAGTAATACACCGTGCAAAAAAAGATGCTAGAAACTGGGCTAGCATAGCTTCTGTGTCTGAGTCTACAGCTATTGTTCCTGGACCACTACGAGTGTTTGACATCCGCAGGGCAGCAGACACTATGAAAAGGATGCAGGGAACAAAGCTAAAAAAACTACTGAAGAACAGACTTGTTTATATTGGCGGATTTGCAAAAAAAGAGAAAACCATAAAAGAAGTTACACAGGATCTGGAAGAACTCCTATGCTGTATTGAAACTATTGACCCAAAAGTAAAGGAAGA |
|  | 11 | AATGACCTCTAGCTCAAGGTTTAATGTGACAAGCTTTTATGCTATTAGGCCAACAAACTCTCATGATGACACAAACATATCTGAGATATTGTCCTATGGTATACAAGAGGGTAAATATGTTAGTATCAAACAGCAAACTATTGACTATTCAACTTACTCAGAGAGGTMCTT |
|  | 12 | ACTGAAGGCATTATAAGTATACTGGATTGTGGGTTGAATGATTTCTATGATTCTCTGGTTGCACTGTTCGGCAACACTACTTTAGCAGACACACTAGACATGGAACAATACTCTACACATAAGGCTGATGACAGGAGAAGCTGTTCTTACCTTTCTATGTTTGCTCTAAATGCACAGGCCTTACAAGCGAAAGTCCCTTACACAGGATATCTGCAGAGCTCCGGCTCAACAAGAGGAGATCAAGCTAGCTCAGGCAACTTTACATTGGCTAGTGCAGAAGGAAATGCAGTGGGTGTCTTTCGTGATGGTATGCTACACATCCATATAGACCATGACTCACCTATCTTGATGACTGAGCTGGCTATTAGAGTTCTAACCTGGGTTACAGGACATGATCATAAAAGTCTTGATAGGAATTCATGTGAAGAATTCCTTAGGCTGTTGCCCAGGTGCAAAAAAAA |
|  | 13 | ACTGAAGAGACCATTAGGCCAATTTCAAGGAAACCTCAGAAAGCCAAGTTGCCAAAAGATATTGAGCATGTTGCTGATAAAGTCCGCAGACAATTATTGGCACAATATAACTCAGACTACATGGGAGATTGTGATAGAATTGTAAGACAGTGTTATGATGGGCACCAGTGTGTACACCACTATAACCTAGGAGCCATAAGCCTAATGAAAACCTTCTTAAGTAGCCAACC |
|  | 14 | TCAGATAGGTTACTCCAGGCAATCACTAGCCGTAGTATTATATGGGGCTTGGCAGGTGGCCTTAAAGAGCTATCTATACCCATATACTCGATATTCTTCAAGGCTTATTTCTTTATAGATAGGACAGATATATGCTCATCTAACAAGTGGGTGTCTATCAGACATGAAGGGCATATGGACAGTTCTGCACAACCATTAGGTACACAAGTTAGAACTAAATTTGGAGTTTGGCTAGACAAAATCTTTCAATGTCCAATGCATTCTGAAATGATGGGTGCATGCCTGGTACTTGATGACAATGCTAGAACATGTAAGGTGGTTCAAGCCAGTTATACAGAGAATGGAGAAGAGACTGAGCTCCATTACATTGCCATTGAT |
|  | 15 | GCAGATATCGTGGGGGTCTCAAAGGTTAGACTGTTTAGTTGCGGATCAGCCTCAAAGGTGGCAAATAATCCAGCAGTAGTGATAGCTTACAGGCTTTGTCCTGAGGCAGTATACCGGCTAAAACCAAGGGGCATCAACTATAGTTCATTAGAGACTGAGGGAACTCACATTGAGGATATTCATCCTGCAATTAGAAGTGAAATCCTTAAGATTATAGCCATGCACAGTTCAGGTGAGGCATTTGCAAGAGAAATTGCACAAGAGAAAATCAGGATGCTTACARCAGCTCTGCCGATATCGACCTCTAGCTCAAGGTTTAATGTGACAAGCTTTTATGCTATTAGGCCAACAAACTCTCATGATGACACAAACATATCTGAGATATTGTCCTATGGTATACAAGAGGGTAAATATGTTAGTATCAAACAGCAAACTATTGACTATTCAACTTACTCAGAGAGGTACTTCATCATCATTGAAGCAATCAGTGTTATCAATCACTTACCCTATGAGGATGATGTAAAGTCTAGATTGATGCAGAACTTCTTGACCTGGGTTCCCACAACTGAAGGCATTATAAGTATACTGGATTGTGGGTTGAATGATATCTGCAGAGCTCCGGTTGCACTGTTCGGCAACACTACATTAGCAGACACACTAGAC |
|  | 16 | GCAGATATCGTGGGGGTCTCAAAGGTTAGACTGTTTAGTTGCGGATCAGCCTCAAAGGTGGCAAATAATCCAGCAGTAGTGATAGCTTACAGGCTTTGTCCTGAGGCAGTATACCGGCTAAAACCAAGGGGCATCAACTATAGTTCATTAGAGACTGAGGGAACTCACATTGAGGATATTCATCCTGCAATTAGAAGTGAAATCCTTAAGATTATAGCCATGCACAGTTCAGGTGAGGCATTTGCAAGAGAAATTGCACAAGAGAAAATCAGGATGCTTACARCAGCTCTGCCGAtATcGACCTCTAGCTCAAGGTTTAATGTGACAAGCTTTTATGCTATTAGGCCAACAAACTCTCATGATGACACAAACATATCTGAGATATTGTCCTATGGTATACAAGAGGGTAAATATGTTAGTATCAAACAGCAAACTATTGACTATTCAACTTACTCAGAGAGGTACTTCATCATCATTGAAGCAATCAGTGTTATCAATCACTTACCCTATGAGGATGATGTAAAGTCTAGATTGATGCAGAACTTCTTGACCTGGGTTCCCACAACTGAAGGCATTATAAGTATACTGGATTGTGGGTTGAATGATTTCTATGATTCTCTGGTTGCACTGTTCGGCAACACTACTTTAGCAGACACACTAGACATGGAACAATACTCTACACATAAGGCTGATGACAGGAGAAGCTGTTCTTACCTTTCTATGTTTGCTCTAAATGCACAGGCCTTACAAGCGAAAGTCCCTTACACAGGATATCTGCAGAGCTCCGGCTCAACAAGAGGAGATCAAGCTAGCTCAGGCAACTTTACATTGGCTAGTGCAGAAGGAAATGCAGTGGGTGTCTTTCGTGATGGTATGCTACACATCCATATAGACCATGACTCACCTATCTTGATGACTGAGCTGGCTATTAGAGTTCTAACCTGGGTTACAGGACATGATCATAAAAGTCTTGATAGGAATTCATGTGAAGAATTCCTTAGGCTGTTGCCCAGGTGCAAAAAAAACACTGCTGTCTCACACGAAGATGATCCGGAGCTCTGCAGATATCTTTCCACTATACCAATTTACCTACACCCAGTTCAGGGTTTCCACAGAAAAACACCATACATCCGACTAAAGAGAAACATCKKARMTcTRCCCAATCGAGAAAGAACAACCTAAGCAATCCATCAACTGTTCATGGCAGCCCGGTAAGCTTGTAATGTACTACCCTTTAATTTTACAAGAAATGCAACCATCTGGCTCAACACTCCAAAGCTTAGAAATTTTGAGAAAGTCTGGCCTGAACTCAGAGTACAATCAACTAAGAAAAGAAGGTGCTCAGAGCTCAAGAAAAGTGGTCATAGCAACAATTAAGCTCAATAGAGATGTTAGTTTGCGCTCAGTTGCACTCCTTCACATGTTCCTTAACCACCTTTCTGGCTTTAAGTCCTACTCATTGGGTGTTCCAGAAAGGGAAGCAGTTCTCCAGAGAATGGTTACCCAGACCAG |
|  | 17 | TATGGTATACAAGCGGGTAAATATGTTAGTATCAAACAGCAAACTATTGACTATTCAACTTACTCAGAGAGGTACTTCATCATCATTGAAGCAATCAGTGTTATCAATCAC |
|  | 18 | CATAGAAAGGTAAGAACAGCTTCTCCTGTCATCAGCCTTATGTGTAGAGTATTGTTCCATGTCTAGTGTGTCTGCTAAAGTAGTGTTGCCGAACAGTGCAACCAGAGAATCATAGAAATCATTCAACCCACAATCCAGTATACTTATAATGCCTTCAGT |
|  | 19 | TTCCACTATACCAATTTACCTACACCCAGTTCAGGGTTTCCACAGAAAAACACCATACATCCGACTAAAGAGAAACATCGGAGCTCTGCCCAATCGAGAAAGAACAACCTAAGCAATCCATCAACTGTTCATGGCAGCCCGGTAAGCTTGTAATGTACTACCCTTTAATTTTACAAGAAATGCAACCATCTGGCTCAACACTCCAAAGCTTAGAAATTTTGAGAAAGTCTGGCCTGAACTCAGAGTACAATCAACTAAGAAAAGAAGGTGCTCAGAGCTCAAGAAAAGTGGTCATAGCAACAATTAAGCTCAATAGAGATGTTAGTTTGCGCTCAGTTGCACTCCTTCACATGTTCCTTAACCACCTTTCTGGCTTTAAGTCCTACTCATTGGGTGTTCCAGAAAGGGAAGCAGTTCTCCAGAGAATGGTTACCCAGACCAG |

**Table S4.** The typical viruses in the order *Bunyavirales* used for phylogenetic analysis.

| Species | Strain | Genus | GenBank accession no. | |
| --- | --- | --- | --- | --- |
|  |  |  | L | S |
| Leopards Hill virus | 11SB17 | *Nairovidae* | NC025831 | AB842090 |
| Leopards Hill virus | 11SB19 | *Nairovidae* | AB842094 | AB842096 |
| Leopards Hill virus | 11SB23 | *Nairovidae* | AB842091 | AB842093 |
| Kasokero virus | Z-52963 | *Nairovidae* | NC036636 | NC029932 |
| Yogue virus | DakAnD56 | *Nairovidae* | KR537453 | KR537455 |
| Chim virus | LEIV-858Uz | *Nairovidae* | KP792711 | KP792713 |
| Bandia virus | RV611 | *Nairovidae* | KU925446 | KU925448 |
| Qalyub virus | ErAg370 | *Nairovidae* | NC034511 | NC034512 |
| Geran virus | LEIV-10899Az | *Nairovidae* | KP792714 | KP792716 |
| Gossas virus | DakAnD401 | *Nairovidae* | KR534878 | KR534876 |
| Uzun Agach virus | LEIV-Kaz155 | *Nairovidae* | KP792741 | KP792743 |
| Issyk-Kul virus | LEIV-315K | *Nairovidae* | KF892055 | KF892057 |
| Issyk-Kul virus | LEZ86-787 | *Nairovidae* | KR537441 | KR537443 |
| Issyk-Kul virus | LEIV-315K | *Nairovidae* | KR709221 | KR709219 |
| Dera Ghazi Khan virus | JD154 | *Nairovidae* | KU925452 | KU925454 |
| Dera Ghazi Khan virus | JD254 | *Nairovidae* | KU343151 | NC034521 |
| Abu Mina virus | EGAN4996 | *Nairovidae* | KU925437 | KU925439 |
| Abu Hammad virus | Art1194 | *Nairovidae* | KU925434 | KU925436 |
| Sapphire II virus | 52301-14 | *Nairovidae* | KU343163 | KU343165 |
| Soldado virus | TRVL52214 | *Nairovidae* | KU925488 | KU925490 |
| Soldado virus | TR52214 | *Nairovidae* | KP792723 | KP792725 |
| Great Saltee virus | RML59972-6 | *Nairovidae* | KU925467 | KU925469 |
| Caspiy virus | LEIV-63Az | *Nairovidae* | KP792708 | KP792710 |
| Zirqa virus | A2070-1 | *Nairovidae* | KU343169 | KU343171 |
| Punta Salinas virus | CalAr888 | *Nairovidae* | KU343157 | KU343159 |
| Punta Salinas virus | CalArt888 | *Nairovidae* | KU925473 | KU925475 |
| Raza virus | 829 | *Nairovidae* | KU925479 | KU925481 |
| Hughes virus | DT-1 | *Nairovidae* | KP792738 | KP792740 |
| Farallon virus | CalAr846 | *Nairovidae* | KU343154 | NC034503 |
| Farallon virus | CalfAr846 | *Nairovidae* | KU925461 | KU925463 |
| Avalon virus | CanAr173 | *Nairovidae* | KU925440 | KU925442 |
| Avalon virus | CanAr173 | *Nairovidae* | KU343145 | NC040458 |
| Tillamook virus | RML86 | *Nairovidae* | KU925494 | KU925496 |
| Taggert virus | Ml14850 | *Nairovidae* | KU925491 | KU925493 |
| Taggert virus | MI14850 | *Nairovidae* | KU343166 | KU925493 |
| Artashat virus | LEIV-9000Az | *Nairovidae* | KP792702 | KP792704 |
| Artashat virus | LEIV-2366Arm | *Nairovidae* | KP792699 | KP792701 |
| Erve virus | Brest/An 221 (TVP21049) | *Nairovidae* | KU925458 | KU925460 |
| Thiafora virus | AnD11411 | *Nairovidae* | NC039220 | NC039222 |
| Thiafora virus | AnD11411 | *Nairovidae* | KR537450 | KR537452 |
| Crimean-Congo hemorrhagic fever virus | Afg09-2990 | *Nairovidae* | HM452307 | HM452305 |
| Crimean-Congo hemorrhagic fever virus | SPU 498/88 | *Nairovidae* | FJ435382 | KJ682820 |
| Crimean-Congo hemorrhagic fever virus | SPU 18/88 | *Nairovidae* | KJ682803 | KJ682818 |
| Kupe virus | K611 | *Nairovidae* | EU257628 | EU257626 |
| Dugbe virus | ArD 44313 | *Nairovidae* | NC004159 | NC004157 |
| Hazara virus | JC280 | *Nairovidae* | NC038709 | KC344857 |
| Hazara virus | JC280 | *Nairovidae* | KP406723 | KP406725 |
| Tofla virus | Toku_Hfla_2013 | *Nairovidae* | LC008512 | LC008510 |
| Tofla virus | Toku_Hfla_2013 | *Nairovidae* | NC029124 | NC029122 |
| Nairobi sheep disease virus | Jilin | *Nairovidae* | NC034387 | NC034386 |
| Nairobi sheep disease virus | 708 | *Nairovidae* | HQ286601 | AF504293 |
| Nairobi sheep disease virus | Ganjam G619 | *Nairovidae* | EU697949 | AF504294 |
| Nairobi sheep disease virus | 779 | *Nairovidae* | HM991306 | HQ286602 |
| Huangpi Tick Virus 1 | H124-1 | *Nairovidae* | KM817667 | KM817734 |
| Huangpi Tick Virus 1 | H124-1 | *Nairovidae* | NC031135 | NC031137 |
| Tamdy virus | LEIV-1308Uz | *Nairovidae* | KP792726 | KP792728 |
| Tamdy virus | LEIV-10226Az | *Nairovidae* | KP792732 | KP792734 |
| Tamdy virus | LEIV-6158Ar | *Nairovidae* | KP792729 | KP792731 |
| Tacheng Tick Virus | TC253 | *Nairovidae* | KM817683 | KM817743 |
| Burana virus | 760 | *Nairovidae* | KP792705 | KP792707 |
| Wenzhou Tick Virus | TS1-2 | *Nairovidae* | NC031291 | KM817745 |
| Wenzhou Tick Virus | TS1-2 | *Nairovidae* | KM817685 | NC031289 |
| South Bay virus isolate | SBV-H-1 | *Nairovidae-like* | KJ746877 | KJ746878 |
| South Bay virus isolate | SC1 | *Nairovidae-like* | KX184198 | KX184199 |
| South Bay virus isolate | H38 | *Nairovidae-like* | KM048320 | KM048321 |
| Pustyn virus | IxRic1 | *Nairovidae-like* | KT007142 | KT007143 |
| Norway nairovirus 1 | B1V | *Nairovidae-like* | MF141048 | MF141049 |
| Norway nairovirus 1 | S5 | *Nairovidae-like* | MF141044 | MF141045 |
| Norway nairovirus 1 | H3 | *Nairovidae-like* | MF141040 | MF141041 |
| Grotenhout virus | Gierle-1 | *Nairovidae-like* | KY700684 | KY700683 |
| Wuhan Millipede Virus 2 | WHWG03 | *Wupederividae* | KM817696 | KM817757.2 |
| Wuhan Millipede Virus 2 | WHWG03 | *Wupederividae* | KM817696 | KM817757.2 |
| Gouleako virus strain | A5/CI/2004 | *Phenuividae* | HQ541738 | HQ541736 |
| Cumuto virus strain | TR7904 | *Phenuividae* | KF543244 | KF543246 |
| Leptomonas moramango leishbunyavirus | LepmorLBV1b-L | *Leishbiviridae* | KX280015 | KX280017 |
| Leptomonas moramango leishbunyavirus | LepmorLBV1a-L | *Leishbiviridae* | KX280012 | KX280014 |
| Wenling frogfish arenavirus 1 | XYHYG11303 | *Arenaviridae* | MG599863 | NC040427 |
| Wenling frogfish arenavirus 2 | XYHYG24857 | *Arenaviridae* | MG599866 | NC040464 |
| Hubei myriapoda virus 5 | GCM10499 | *Myponviridae* | NC033761 | NC033760 |
| Hubei myriapoda virus 5 | WGML140132 | *Myponviridae* | KX884840 | KX884842 |
| Hubei myriapoda virus 5 | GCM10499 | *Myponviridae* | KX884758 | KX884760 |
| Hantavirus | L99 | *Hantanviridae* | AF288297 | AF288299 |
| Bruges hantavirus | BE/Vieux-Genappe/TE/2013/1 | *Hantanviridae* | KX551962 | NC034394 |
| Maripa hantavirus | BOR | *Hantanviridae* | JQ611713 | JQ611712 |
| Wuchang Cockraoch Virus 1 | WCZL-5 | *Phamaviridae* | NC031293 | NC031296 |
| Wuhan Mosquito Virus 1 | WT3-15 | *Phamaviridae* | NC031307 | NC031310 |
| Kigluaik phantom virus | G10N | *Phamaviridae* | NC034462 | NC034463 |
| Wuhan Mosquito Virus 2 | QN2-7 | *Wupederividae* | KM817698 | KM817759 |
| Groundnut bud necrosis virus | Guntur chilli | *Tospoviridae* | KX965705 | KX279884 |
| Watermelon silver mottle virus | DD6 S RAN | *Tospoviridae* | NC003832 | AY864852 |
| Impatiens necrotic spot virus | Pepe | *Tospoviridae* | LC384870 | LC384872 |
| Tomato spotted wilt virus | TSWV-LE | *Tospoviridae* | KU976394 | KU976396 |
| Actinidia chlorotic ringspot associated virus | HN-6 | *Fimoviridae* | KT861481 | NC038772 |
| Redbud yellow ringspot virus |  | *Fimoviridae* | NC038852 | NC038854 |
| European mountain ash ringspot-associated virus | E52165 | *Fimoviridae* | AY563040 | HG799708 |
| Pigeonpea sterility mosaic virus 2 | PLant | *Fimoviridae* | NC030660 | HF912245 |
| Herbert virus | F23/CI/2004 | *Perbunyaviridae* | NC038714 | NC038712 |
| Kibale virus | P05/UG/2008 | *Perbunyaviridae* | NC034460 | KF590575 |
| Wenling crustacean virus 9 | WLJQ100911 | *Cruliriviridae* | KX884856 | KX884858 |
| Wenling crustacean virus 9 | WLJQ100911 | *Cruliriviridae* | NC032143 | NC032145 |

**Table S5.** Amino acid sequence similarity (%) between BJNV and the members in the family *Nairoviridae* or other nairo-like viruses^*^.

| Protein | Virus | BJNV HLJ2015 | GTV | NNV-1 | PTV | GTHV | SBV | ATSV | CCHFV | DBV | NSDV |
| --- | --- | --- | --- | --- | --- | --- | --- | --- | --- | --- | --- |
| RdRp |  |  |  |  |  |  |  |  |  |  |  |
|  | BJNV YKS44 | 97.9 | 97.2 | 83.8 | 83.7 | 83.3 | 56.6 | 22.3 | 22.1 | 21.1 | 21.3 |
|  | BJNV HLJ2015 | *** | 97.5 | 83.9 | 84.1 | 83.8 | 56.6 | 22.3 | 22.1 | 21.2 | 21.2 |
|  | GTV | *** | *** | 84.3 | 84.1 | 83.8 | 56.7 | 22.4 | 22.3 | 21.2 | 21.3 |
|  | NNV-1 | *** | *** | *** | 98.3 | 97.4 | 56.9 | 22.5 | 22.4 | 21.1 | 21.7 |
|  | PTV | *** | *** | *** | *** | 97.7 | 57 | 22.6 | 22.3 | 21.1 | 21.8 |
|  | GTHV | *** | *** | *** | *** | *** | 56.6 | 22.3 | 22.1 | 20.9 | 21.6 |
|  | SBV | *** | *** | *** | *** | *** | *** | 22.4 | 28.1 | 26.4 | 27.2 |
|  | ATSV | *** | *** | *** | *** | *** | *** | *** | 56.8 | 55.4 | 56.3 |
|  | CCHFV | *** | *** | *** | *** | *** | *** | *** | *** | 68 | 70.6 |
|  | DBV | *** | *** | *** | *** | *** | *** | *** | *** | *** | 72.9 |
| NP |  |  |  |  |  |  |  |  |  |  |  |
|  | BJNV YKS44 | 99.5 | 99.5 | 85.7 | 86.5 | 85.6 | 57 | 21.2 | 19.6 | 18.5 | 18.9 |
|  | BJNV HLJ2015 | *** | 98.6 | 85.7 | 86.5 | 85.6 | 57.2 | 21.4 | 19.6 | 18.5 | 18.9 |
|  | GTV | *** | *** | 85.7 | 86.5 | 85.6 | 57.2 | 21.4 | 19.6 | 18.5 | 18.9 |
|  | NNV-1 | *** | *** | *** | 98.4 | 98.8 | 57.4 | 20.3 | 19.6 | 17.8 | 18.7 |
|  | PTV | *** | *** | *** | *** | 98.8 | 57.4 | 20.5 | 19.4 | 17.6 | 18.4 |
|  | GTHV | *** | *** | *** | *** | *** | 57.6 | 20.1 | 19.4 | 17.8 | 18.5 |
|  | SBV | *** | *** | *** | *** | *** | *** | 19.8 | 16.4 | 16.9 | 18.4 |
|  | ATSV | *** | *** | *** | *** | *** | *** | *** | 38.7 | 37.3 | 38.1 |
|  | CCHFV | *** | *** | *** | *** | *** | *** | *** | *** | 54.4 | 57.8 |
|  | DBV | *** | *** | *** | *** | *** | *** | *** | *** | *** | 56.1 |

^*^ BJNV, Beiji nairovirus; GTV, Gakugsa tick virus; NNV-1, Norway nairovirus 1; PTV, Pustyn virus; GTHV, Grotenhout virus; SBV, South Bay virus; ATSV, Artashat virus; CCHFV, Crimean-Congo hemorrhagic fever virus; DBV, Dugbe virus; NSDV, Nairobi sheep disease virus; RdRp, RNA dependent RNA polymerase; NP, nucleoprotein

**Table S6.** Nucleotide sequence similarity (%) between BJNV and the members in the family *Nairoviridae* or other nairo-like viruses^*^.

| Protein | Virus | BJNV HLJ2015 | GTV | NNV-1 | PTV | GTHV | SBV | ATSV | CCHFV | DBV | NSDV |
| --- | --- | --- | --- | --- | --- | --- | --- | --- | --- | --- | --- |
| RdRp |  |  |  |  |  |  |  |  |  |  |  |
|  | BJNV YKS44 | 97.8 | 96.6 | 78.3 | 78.1 | 78.3 | 58.8 | 36.7 | 39.1 | 40 | 39.4 |
|  | BJNV HLJ2015 | *** | 97.2 | 78.3 | 78.2 | 78.3 | 58.8 | 36.7 | 38.5 | 39.7 | 39.4 |
|  | GTV | *** | *** | 78.4 | 78.4 | 78.3 | 59 | 36.7 | 38.5 | 39.5 | 39.5 |
|  | NNV-1 | *** | *** | *** | 97.7 | 96.7 | 59.2 | 33.2 | 38.7 | 39.9 | 38.9 |
|  | PTV | *** | *** | *** | *** | 97.3 | 59.1 | 32.8 | 38.4 | 39.8 | 39 |
|  | GTHV | *** | *** | *** | *** | *** | 58.9 | 33 | 38.3 | 39.7 | 42.3 |
|  | SBV | *** | *** | *** | *** | *** | *** | 40.3 | 43.8 | 44.7 | 43.8 |
|  | ATSV | *** | *** | *** | *** | *** | *** | *** | 62.4 | 61.4 | 62 |
|  | CCHFV | *** | *** | *** | *** | *** | *** | *** | *** | 67.5 | 68.5 |
|  | DBV | *** | *** | *** | *** | *** | *** | *** | *** | *** | 70.3 |
| NP |  |  |  |  |  |  |  |  |  |  |  |
|  | BJNV YKS44 | 99.1 | 98 | 80.2 | 80 | 80.1 | 60.9 | 35 | 33.9 | 36.6 | 34.1 |
|  | BJNV HLJ2015 | *** | 97.9 | 79.7 | 79 | 79.9 | 60.9 | 35 | 33.9 | 36.6 | 34.1 |
|  | GTV | *** | *** | 80 | 80 | 79.9 | 60.8 | 35.2 | 33.7 | 36.5 | 34.1 |
|  | NNV-1 | *** | *** | *** | 97.7 | 98.7 | 61.3 | 35.8 | 34.4 | 36 | 34.8 |
|  | PTV | *** | *** | *** | *** | 97.8 | 61.1 | 35.5 | 34.8 | 36.2 | 34.8 |
|  | GTHV | *** | *** | *** | *** | *** | 61.2 | 35.7 | 34.4 | 36 | 34.8 |
|  | SBV | *** | *** | *** | *** | *** | *** | 35.9 | 35.4 | 35.9 | 35.4 |
|  | ATSV | *** | *** | *** | *** | *** | *** | *** | 57.7 | 55.4 | 55.3 |
|  | CCHFV | *** | *** | *** | *** | *** | *** | *** | *** | 66.9 | 68.2 |
|  | DBV | *** | *** | *** | *** | *** | *** | *** | *** | *** | 67.1 |

^*^ BJNV, Beiji nairovirus; GTV, Gakugsa tick virus; NNV-1, Norway nairovirus 1; PTV, Pustyn virus; GTHV, Grotenhout virus; SBV, South Bay virus; ATSV, Artashat virus; CCHFV, Crimean-Congo hemorrhagic fever virus; DBV, Dugbe virus; NSDV, Nairobi sheep disease virus; RdRp, RNA dependent RNA polymerase; NP, nucleoprotein

**Table S7.** Information on the studied populations in this study.

| Epidemiology character | Patients with BJNV (n=67) | Hospitalized patients (n=658) | Control population (n=100) |
| --- | --- | --- | --- |
| Region |  |  |  |
| Inner Mongolia | 49 (73.1) | 470 (71.4) | 60 (60.0) |
| Heilongjiang | 18 (26.9) | 179 (27.2) | 40 (40.0) |
| Liaoning | 0 | 9 (1.4) | 0 |
| Year |  |  |  |
| 2017 | 43 (64.2) | 348 (52.9) | 50 (50.0) |
| 2018 | 24 (35.8) | 310 (47.1) | 50 (50.0) |
| Age |  |  |  |
| <40 | 12 (17.9) | 151 (22.9) | 18 (18.0) |
| 40-60 | 52 (77.6) | 445 (67.6) | 75 (75.0) |
| >60 | 3 (4.5) | 62 (9.5) | 7 (7.0) |
| Sex |  |  |  |
| Male | 47 (70.1) | 450 (68.4) | 65 (65.0) |
| Female | 20 (29.9) | 208 (31.6) | 35 (35.0) |
| Month distribution |  |  |  |
| Before May | 16 (23.9) | 65 (9.9) | 7 (7.0) |
| May to July | 44 (65.7) | 537 (81.6) | 78 (78.0) |
| After July | 7 (10.4) | 56 (8.5) | 15 (15.0) |
| Profession |  |  |  |
| Field worker^*^ | 67 (100) | 638 (97.0) | 0 |
| others | 0 | 20 (3.0） | 100 (100.0) |
| Tick bite history |  |  |  |
| Yes | 67 (100.0) | 650 (98.8) | 0 (0) |
| No | 0 | 0 | 100 (100.0) |
| nd | 0 (0.0) | 8 (1.2) | 0 (0) |

^*^Field worker include farmer, forestry worker, or other people who living in wooded and hilly areas and working in fields.

**Table S**8. Serological detection of BJNV-infected patients.

| Patient No. | Sex | Age (year) | Province | Sampling time (days after tick bite) | ELISA* | |
| --- | --- | --- | --- | --- | --- | --- |
|  |  |  |  |  | IgG | IgM |
| H18-14 | F | 44 | Inner Mongolia | 7 | <20 | 40 |
| H18-39 | M | 52 | Inner Mongolia | 15 | <20 | <20 |
| H18-44 | M | 46 | Inner Mongolia | 10 | <20 | 20 |
| H18-56 | M | 29 | Inner Mongolia | 10 | 40 | 20 |
| H18-59 | M | 30 | Inner Mongolia | 6 | <20 | 80 |
| H18-64 | F | 35 | Inner Mongolia | 7 | <20 | <20 |
| H18-93 | M | 28 | Inner Mongolia | 21 | <20 | <20 |
| H18-122 | F | 46 | Inner Mongolia | 11 | <20 | 20 |
| H18-160 | F | 44 | Inner Mongolia | 4 | <20 | 40 |
| H18-316 | F | 50 | Inner Mongolia | 30 | 40 | <20 |
| H18-322 | M | 48 | Inner Mongolia | 35 | 20 | 20 |
| H18-639 | M | 54 | Inner Mongolia | 10 | <20 | <20 |
| H18-790 | M | 56 | Inner Mongolia | 41 | 160 | <20 |
| H18-801 | F | 42 | Inner Mongolia | 11 | <20 | 20 |
| H18-807 | M | 51 | Heilongjiang | 11 | 160 | 20 |
| H18-857 | F | 55 | Inner Mongolia | 15 | 80 | 20 |
| H18-970 | M | 45 | Inner Mongolia | 15 | <20 | <20 |
| H18-974 | M | 63 | Inner Mongolia | 15 | <20 | 20 |
| H18-1063 | M | 36 | Inner Mongolia | 41 | 80 | 20 |
| H18-1199 | F | 54 | Inner Mongolia | 31 | 20 | 40 |
| H18-1374 | M | 41 | Heilongjiang | 16 | 20 | 20 |
| H18-1414 | M | 31 | Inner Mongolia | 19 | <20 | <20 |
| H18-1467 | F | 53 | Inner Mongolia | 6 | <20 | 20 |
| H18-1486 | M | 54 | Inner Mongolia | 21 | <20 | <20 |
| H17-115 | F | 40 | Inner Mongolia | 15 | 20 | 20 |
| H17-116 | F | 39 | Inner Mongolia | 8 | <20 | 20 |
| H17-136 | M | 32 | Inner Mongolia | 5 | <20 | 20 |
| H17-155 | M | 38 | Inner Mongolia | 9 | <20 | 20 |
| H17-177 | M | 47 | Inner Mongolia | 11 | 160 | <20 |
| H17-320 | M | 44 | Heilongjiang | 9 | <20 | <20 |
| H17-422 | M | 46 | Inner Mongolia | 8 | <20 | 20 |
| H17-440 | M | 65 | Inner Mongolia | 3 | <20 | 40 |
| H17-449 | M | 52 | Inner Mongolia | 16 | 20 | 20 |
| H17-496 | M | 54 | Heilongjiang | 10 | <20 | <20 |
| H17-497 | M | 43 | Inner Mongolia | 10 | 20 | <20 |
| H17-514 | M | 51 | Inner Mongolia | 9 | <20 | <20 |
| H17-523 | F | 32 | Inner Mongolia | 10 | <20 | <20 |
| H17-572 | M | 50 | Inner Mongolia | 10 | <20 | <20 |
| H17-595 | F | 42 | Heilongjiang | 11 | <20 | <20 |
| H17-617 | F | 35 | Inner Mongolia | 9 | <20 | 40 |
| H17-618 | M | 41 | Heilongjiang | 10 | <20 | 20 |
| H17-641 | M | 51 | Heilongjiang | 21 | <20 | 40 |
| H17-666 | F | 59 | Heilongjiang | 6 | <20 | 20 |
| H17-701 | M | 50 | Inner Mongolia | 5 | <20 | 20 |
| H17-712 | M | 50 | Inner Mongolia | 31 | 160 | <20 |
| H17-809 | M | 73 | Heilongjiang | 11 | <20 | 20 |
| H17-818 | M | 48 | Heilongjiang | 17 | 320 | 20 |
| H17-908 | F | 85 | Inner Mongolia | 15 | 40 | <20 |
| H17-991 | F | 61 | Heilongjiang | 9 | <20 | 20 |
| H17-1004 | M | 44 | Heilongjiang | 7 | <20 | 20 |
| H17-1023 | M | 67 | Inner Mongolia | 15 | 80 | <20 |
| H17-1067 | M | 55 | Heilongjiang | 2 | <20 | 10 |
| H17-1249 | M | 43 | Heilongjiang | 15 | <20 | 20 |
| H17-1263 | M | 53 | Inner Mongolia | 4 | <20 | 20 |
| H17-1265 | M | 47 | Heilongjiang | 10 | <20 | <20 |
| H17-1266 | M | 50 | Heilongjiang | 32 | <20 | <20 |
| H17-1318 | M | 38 | Inner Mongolia | 6 | <20 | 20 |
| H17-1378 | M | 54 | Heilongjiang | 17 | <20 | 20 |
| H17-1440 | M | 45 | Inner Mongolia | 18 | <20 | 20 |
| H17-1451 | M | 50 | Heilongjiang | 30 | 40 | 40 |
| H17-1528 | M | 52 | Inner Mongolia | 41 | <20 | <20 |
| H17-1550 | M | 46 | Inner Mongolia | 9 | <20 | 20 |
| H17-1555 | M | 49 | Inner Mongolia | 2 | <20 | 160 |
| H17-1622 | M | 52 | Inner Mongolia | 45 | 320 | 10 |
| H17-1722 | F | 54 | Heilongjiang | 14 | <20 | <20 |
| H17-1735 | M | 47 | Inner Mongolia | 13 | <20 | 20 |
| H17-1740 | F | 30 | Inner Mongolia | 21 | 20 | <20 |

^*^ The detection methods and result judgement are described in Methods of the text. M, male; F, female; ELISA, enzyme linked immunosorbent assay.

**Table S9. Detection of BJNV RNA in ticks in China by nested PCR^*^.**

| **Tick species** | **No. of pools/No. of ticks tested (%, 95% CI)^§^** | | | |
| --- | --- | --- | --- | --- |
|  | **Heilongjiang** | **Inner Mongolia** | **Jilin** | **Total** |
| *Ixodes persulcatus* | 151/851 (1.3, 0.7-2.8) | 90/165 (4.8, 2.0-10.3) | 58/117 (4.2, 1.4-10.6） | 299/1133 (2.0, 1.3-3.1) |
| *Ixodes crenulatus* | 131/661 (1.5, 0.7-2.73) | 15/30 (3.0, 0.2-20.4) | 75/120 (5.7, 2.3-13.6) | 221/811 (2.1, 1.2-3.4) |
| *Demacentor silvarum* | 0/47 (0) | nd | 28/57 (3.9, 0.8-14.4) | 28/104 (2.1, 0.4-6.9) |
| *Demacentor nuttalli* | 0/83 (0) | nd | 14/149 (0.67, 0.0-3.3) | 14/232 (0.4, 0.03-2.10) |
| *Haemaphyslis conicinna* | 61/403 (1.1, 0.3-2.5) | nd | nd | 61/403 (1.1, 0.3-2.5) |
| *Haemaphysalis longiconis* | 43/694 (0.4, 0.1-1.2) | 0/16 (0) | 0/6 (0) | 43/716 (0.4, 0.1-1.2) |
| Total | 386/2739 (1.0, 0.7-1.8) | 105/211 (4.3, 1.9-8.7) | 175/449 (3.3, 1.8-5.6) | 666/3399 (1.5, 1.1-1.9) |

^*^nd, no data.

^§^The prevalence was expressed as the minimum infection rate that was calculated by maximum likelihood estimation (MLE) using the program PooledInfRat.

**Table S10.** Nucleotide sequences of BJNV amplified in different hosts in northeastern China.

| Sequence ID | GenBank accession no. | Virus isolate | Genome segment* | Sampling site | Host |
| --- | --- | --- | --- | --- | --- |
| Seq1 | MN122079 | YKS44 | L | Inner Mongolia, Yakeshi | Human |
| Seq2 | MN122080 | YKS44 | S | Inner Mongolia, Yakeshi | Human |
| Seq3 | MW315101 | H160 | L | Inner Mongolia, Moqi | Human |
| Seq4 | MW315107 | H160 | S | Inner Mongolia, Moqi | Human |
| Seq5 | MW315102 | H801 | L | Inner Mongolia, Evenk | Human |
| Seq6 | MW315108 | H801 | S | Inner Mongolia, Evenk | Human |
| Seq7 | MW315103 | H39 | L | Inner Mongolia, Evenk | Human |
| Seq8 | MW315109 | H39 | S | Inner Mongolia, Evenk | Human |
| Seq9 | MW315104 | H59 | L | Inner Mongolia, Evenk | Human |
| Seq10 | MW315110 | H59 | S | Inner Mongolia, Evenk | Human |
| Seq11 | MW315105 | H56 | L | Inner Mongolia, Moqi | Human |
| Seq12 | MW315111 | H56 | S | Inner Mongolia, Moqi | Human |
| Seq13 | MW315106 | H1063 | L | Inner Mongolia, Genhe | Human |
| Seq14 | MW315112 | H1063 | S | Inner Mongolia, Genhe | Human |
| Seq15 | MN122083 | YKS322 | L | Inner Mongolia, Yakeshi | Human |
| Seq16 | MN122084 | YKS322 | S | Inner Mongolia, Yakeshi | Human |
| Seq17 | MN122085 | DH58 | L | Jilin, Dunhua | *Ixodes crenulaus* |
| Seq18 | MN122086 | DH58 | S | Jilin, Dunhua | *Ixodes crenulaus* |
| Seq19 | MN122087 | YC854 | L | Heilongjiang, Yichun | *Ixodex persulcatu*s |
| Seq20 | MN122088 | YC854 | S | Heilongjiang, Yichun | *Ixodex persulcatus* |
| Seq21 | MN122089 | DH73 | L | Heilongjiang, Dunhua | *Ixodex persulcatu*s |
| Seq22 | MN122090 | DH73 | S | Heilongjiang, Dunhua | *Ixodex persulcatus* |
| Seq23 | MN122091 | YC908 | L | Heilongjiang, Yichun | *Ixodes crenulaus* |
| Seq24 | MN122092 | YC908 | S | Heilongjiang, Yichun | Ixodes crenulaus |
| Seq25 | MN122093 | YC846 | L | Heilongjiang, Yichun | *Ixodes crenulaus* |
| Seq26 | MN122094 | YC846 | S | Heilongjiang, Yichun | *Ixodes crenulaus* |
| Seq27 | MN122095 | ALS66 | L | Inner Mongolia, Alongshan | *Ixodex persulcatu*s |
| Seq28 | MN122096 | ALS66 | S | Inner Mongolia, Alongshan | *Ixodex persulcatus* |
| Seq29 | MN122097 | SW41 | L | Inner Mongolia, Alongshan | Sheep |
| Seq30 | MN122098 | SW41 | S | Inner Mongolia, Alongshan | Sheep |
| Seq31 | MN122099 | EEGN25 | L | Inner Mongolia, Alongshan | Cattle |
| Seq32 | MN122100 | EEGN25 | S | Inner Mongolia, Alongshan | Cattle |

^*^ L, large segment; S, small segment.

**Table S11.** Laboratory findings in patients with BJNV infection^*^.

|  | **Normal (%)** | **Increased (%)** | **Decreased (%)** | **Results (SD or IQR)** |
| --- | --- | --- | --- | --- |
| **Blood routine** | | | | |
| Leucocytes (×10^9^ per L, normal range 3.5-9.5) | 53 (80.3) | 6 (9.1) | 7 (10.6) | 6.2 (3.0) |
| Neutrophils (×10^9^ per L, normal range 1.6-6.4) | 53 (84.1) | 8 (12.7) | 2 (3.2) | 4.0 (2.2) |
| Lymphocytes (×10^9^ per L, normal range 1.1-3.2) | 44 (69.8) | 0 | 19 (30.2) | 1.4 (0.6) |
| Monocytes (×10^9^ per L, normal range 0.1-0.6) | 44 (69.8) | 19 (30.2) | 0 | 0.5 (0.2) |
| Platelets (×10^9^ per L, normal range 125.0-350.0) | 51 (78.5) | 3 (4.6) | 11 (16.9) | 195.0 (93.8) |
| Haemoglobin (g/L, normal range 131-172) | 49(86.6) | 2 (3.1) | 13 (10.3) | 141.6 (22.5) |
| **Coagulation function** | | | | |
| Activated partial thromboplastin time (s, normal range 19.9-33.3) | 53 (88.3) | 7 (11.7) | 0 | 27.7 (4.3) |
| Prothrombin time (s, normal range 9.7-13.2) | 59 (98.3) | 1 (1.7) | 0 | 11.5 (0.8) |
| D-dimer (μg/L, normal range 0.0-1.5) | 6 (66.7) | 3 (33.3) | 0 | 0.4 (0.2-1.8) |
| **Blood biochemistry** | | | | |
| Albumin (g/L, normal range 40.0-55.0) | 4 (30.8) | 0 | 9 (69.2) | 4 (30.8) |
| Alanine aminotransferase (U/L, normal range 9.0-50.0) | 44 (67.7) | 20 (30.8) | 1 (1.5) | 32.0 (26.0-66.0) |
| Aspartate aminotransferase (U/L, normal range 15.0-40.0) | 38 (58.2) | 25 (38.5) | 2 (3.3) | 29.0 (23.0-67.0) |
| Total bilirubin (μmol/L, normal range 5.0-21.0) | 50 (82.0) | 11 (18.0) | 0 | 15.3 (9.4) |
| Blood urea nitrogen (mmol/L, normal range 3.1-8.8) | 50 (79.4) | 4 (6.3) | 9 (14.3) | 4.9 (1.7) |
| Serum creatinine (μmol/L, normal range 41-97) | 49 (90.5) | 5 (7.9) | 1 (1.6) | 71.8 (14.5) |
| Creatine kinase (U/L, normal range 40-310) | 14 (70.0) | 3 (15.0) | 3 (15.0) | 76.0 (57.0-154.0) |
| Lactate dehydrogenase (U/L, normal range 120-250) | 10 (58.8) | 7 (41.2) | 0 | 306.1 (204.8) |
| Myoglobin (ng/mL, normal range 0.0-150.0) | 8 (47.1) | 4 (23.5) | 5 (29.4) | 29.1 (21.5-85.7) |
| Glucose (mmol/L, normal range 3.9-6.1) | 19 (57.6) | 14 (42.4) | 0 | 6.5 (2.1) |
| Potassium (mmol/L, normal range 3.5-5.3) | 57 (90.5) | 0 | 6 (9.5) | 3.9 (0.3) |
| Sodium (mmol/L, normal range 137-147) | 39 (68.4) | 0 | 18 (31.6) | 138 (3.0) |
| C-reactive protein (mg/L, normal range 0.0-3.0) | 8 (25.6) | 21 (74.4) | 0 | 23.3 (23.8) |

^*^Data are n (%), mean (SD), and median (IQR). Increased means over the upper limit of the normal range and decreased means below the lower limit of the normal range.
